# Integrative revision of the giant pill-millipede genus *Sphaeromimus* from Madagascar, with the description of seven new species (Diplopoda, Sphaerotheriida, Arthrosphaeridae)

**DOI:** 10.3897/zookeys.414.7730

**Published:** 2014-06-06

**Authors:** Thomas Wesener, Daniel Minh-Tu Le, Stephanie F. Loria

**Affiliations:** 1 Field Museum of Natural History, Zoology - Insects, 1400 S. Lake Shore Drive, 60605 Chicago, Illinois, U.S.A.; 2 Zoologisches Forschungsmuseum Alexander Koenig, Leibniz Institute for Animal Biodiversity, Center for Taxonomy and Evolutionary Research (Section Myriapoda), Adenauerallee 160, 53113 Bonn, Germany; 3 School of the Art Institute of Chicago, 36 S. Wabash Avenue, 60603 Chicago, Illinois, U.S.A.; 4 American Museum of Natural History, Richard Glider Graduate School, Central Park West at 79th Street, New York, U.S.A.

**Keywords:** COI, Barcoding, soil arthropods, microendemism, Andrahomana, Lavasoa, Sainte Luce, Manombo

## Abstract

The Malagasy giant pill-millipede genus *Sphaeromimus* de Saussure & Zehntner, 1902 is revised. Seven new species, *S. titanus* **sp. n.**, *S. vatovavy* **sp. n.**, *S. lavasoa* **sp. n.**, *S. andohahela* **sp. n.**, *S. ivohibe* **sp. n.**, *S. saintelucei* **sp. n.**, and *S. andrahomana* **sp. n.** were discovered, in one case with the help of sequence data, in the rainforests of southeastern Madagascar. The species are described using light- and scanning electron microscopy. A key to all 10 species of the genus is presented. All but one (*S. andohahela*) of the newly discovered species are microendemics each occurring in isolated forest fragments. The mitochondrial COI barcoding gene was amplified and sequenced for 18 *Sphaeromimus* specimens, and a dataset containing COI sequences of 28 specimens representing all *Sphaeromimus* species (except *S. vatovavy*) was analyzed. All species are genetically monophyletic. Interspecific uncorrected genetic distances were moderate (4–10%) to high (18–25%), whereas intraspecific variation is low (0–3.5%). Sequence data allowed the correct identification of three colour morphs of *S. musicus*, as well as the identity of a cave specimen, which although aberrant in its morphology and colouration, was genetically identical to the holotype of *S. andrahoma.*

## Introduction

Madagascar, the world’s third largest island, is famous for its endemic and endangered fauna and flora (Myers et al. 2000, Vences et al. 2009). Some representatives of the millipedes, class Diplopoda, represent quite charismatic invertebrate endemics of Madagascar. These include the large-bodied, strikingly red-black colored so-called ‘Fire-Millipedes’ of the order Spirobolida (Wesener et al. 2009a, Wesener et al. 2011a), and the giant pill-millipedes, locally called ‘Tainkintana’ (=star droppings), reaching the size of a small orange or a tennis ball when rolled-up.

Despite their conspicuousness, it was only recently that the millipede biodiversity on Madagascar became better known. Mauriès (1994, 1997) discovered and described the first African representatives of the order Chordeumatida from Madagascar, which belong to the same family Pygmaesomatidae as certain Indian endemics. Inventories of the ‘classical’ millipedes of the order Spirobolida led to the discovery of 13 new genera and 53 new species (Wesener et al. 2008, Wesener and Enghoff 2009, Wesener et al. 2009b). Recently the occurrence of the order Polyzoniida on Madagascar was reviewed, and was found to be diverse and indigenous (Wesener 2014a), while representatives of the order Siphonophorida were discovered on Madagascar for the first time (Wesener in press)

In giant pill-millipedes, all species known from Madagascar were redescribed (Wesener and Sierwald 2005, Wesener and Wägele 2008). New discoveries included the first dwarfed member of the giant pill-millipede order, the genus *Microsphaerotherium* Wesener & VandenSpiegel, 2007, as well as a surge of species in the genus *Zoosphaerium* Pocock, 1895, which now includes 62 described species (Wesener 2009), including the largest Sphaerotheriida by far, e.g. *Zoosphaerium neptunus* (Butler, 1872) reaching the size of a tennis ball or small orange when rolled-up.

The third Malagasy giant pill-millipede genus *Sphaeromimus* de Saussure & Zehntner, 1902, is an unusual representative of the order. One characteristic of the genus is the presence of well-developed stridulation organs, the male ‘harp’ and the female ‘washboard’, which carry more stridulation ribs than in any other member of the Sphaerotheriida. These stridulation organs are still not well understood, but may play a role during courtship (Wesener et al. 2011b). The first species, *Sphaeromimus musicus* was described in 1897 (de Saussure and Zehntner 1897), with no additional specimens found for more than 100 years. The unusual morphology of *Sphaeromimus*, quite distinct from the majority of Malagasy giant pill-millipedes belonging to the genus *Zoosphaerium* Pocock, 1895, let the experts to suggest that *Sphaeromimus musicus* might represent a “mislabelled or an introduced Indian sphaerotheriid” (Jeekel 1999). However, a century later, two additional species of *Sphaeromimus* were discovered in littoral rainforest fragments in southeastern Madagascar, and its type species could be redescribed based on numerous samples taken from the southern Malagasy spiny forest ecosystem during general biodiversity inventory programs (Wesener and Sierwald 2005). A phylogenetic analysis of the Sphaerotheriida based on morphological (Wesener and VandenSpiegel 2009, Wesener 2014b) as well as molecular characters (Wesener et al. 2010) confirmed the sister-group relationship of *Sphaeromimus* to the Indian genus *Arthrosphaera* Pocock, 1895, the first time such a Madagascar-India relationship was discovered in soil arthropods. *Sphaeromimus* is more closely related to the Indian genus *Arthrosphaera* than to the other Malagasy giant pill-millipede genera *Zoosphaerium* and *Microsphaerotherium*, all of which belong to the family Arthrosphaeridae (Wesener 2014b).

An expedition to Madagascar conducted by TW in 2007, as well as sorting through different natural history collections, led to the discovery of 12 additional *Sphaeromimus* populations representing seven undescribed species of *Sphaeromimus*, all from humid forests in southeastern Madagascar. Many of the newly discovered species were only found in tiny rainforest vestiges/fragments, and one specimen was discovered in a cave located in the southern dry spiny forest ecosystem. While all known *Sphaeromimus* show a ‘normal’ size of 18–35 mm, one of the undescribed species shows gigantism. These findings highlight how little we still know about the biodiversity of one of the most striking invertebrate endemics on Madagascar.

## Methods

### Specimen collecting and conservation

*Sphaeromimus* specimens were collected by hand. Three of the seven newly discovered species were found in natural history collections: one historic and two obtained during general arthropod inventory programs on Madagascar. Specimens of the other four species were collected from eight localities during an expedition by TW and Kai Schütte (University of Hamburg). Between 12–20 hours were spent searching at each locality. Rarely were the *Sphaeromimus* individuals encountered in high numbers (spiny forest, Sainte Luce S9, Grande Lavasoa) and usually several hours of search were necessary to find an area where 3–5 specimens could be collected. The isolated occurrence patterns of *Sphaeromimus* species might be the main reason why no *Sphaeromimus* specimens were collected during general inventory programs, which targeted the same localities as we did in 2007. Legs were removed from several specimens and placed in 95% ethanol for DNA analysis, while the rest of the specimens was preserved in 80% ethanol, which was changed twice. A few months later, some of the specimens were transferred to 95% ethanol to facilitate future DNA work on the specimens.

### Illustrations

Important structures of the *Sphaeromimus* specimens were drawn using a *camera lucida* mounted on an Olympus SZX12 stereo-microscope. Pencil drawings were later transferred to ink with pigma micron pens. For scanning electron microscopy, samples were dehydrated via an ethanol series (90%, 95%, 2× 100%), dried over night, and mounted on aluminium stubs before being sputter coated with gold. SEM images were taken using a Zeiss Leo EVO SEM (FMNH) and a Hitachi S-2460 SEM (ZFMK). All images were later modified using Adobe Photoshop CS2 and assembled into plates using Adobe Illustrator CS2.

### DNA extraction, sequencing

DNA was extracted from 18 specimens: 12 of them preserved in 95% ethanol, the remaining ones in 75% ethanol. The HCO/LCO primer pair (Folmer et al. 1994) was used to sequence a 674 bp fragment of the mitochondrial cytochrome *c* oxidase subunit I (COI) gene. DNA extraction, PCR, purification, and sequencing protocols were identical to those used in a previous study (Wesener et al. 2010). While the COI gene, being a mitochondrial gene as well as containing little resolution at deeper evolutionary splits, does not allow a reconstruction of the phylogeny of the *Sphaeromimus* species, we aimed at finding a unique identifier allowing us to study and illustrate the genetic distances between the different species of the genus. All obtained sequences were checked via Blast searches (Altschul et al. 1997), no contaminations were discovered. The sequences were aligned by hand in BioEdit (Hall 1999) with those obtained during a previous study (Wesener et al. 2010) from other *Sphaeromimus* specimens, using as outgroup taxa a specimen of the basal family Procyliosomatidae (Wesener and VandenSpiegel 2009), as well as two species of the other Malagasy genus *Zoosphaerium*, including the type *Zoosphaerium neptunus*, and a member of the closely related Indian genus *Arthrosphaera*. All newly sequenced *Sphaeromimus* sequences were uploaded to Genbank (Accession #: KJ13244–KJ13261, see Table 1).

**Table 1.** *Sphaeromimus* samples, Genbank code, and depository. Genbank numbers marked by an asterisk (*) were published in a previous study (Wesener et al. 2010).

Species	Specimen Catalog #	Locality	Genbank #	GenSeq
*Procyliosoma leae* *	QVMAG 23:45801	Tasmania	FJ409910*	genseq-4
Zoosphaerium neptunus*	FMNH-INS 56005	Madagascar, Andasibe	FJ409929*	genseq-4
Zoosphaerium alluaudi*	FMNH-INS 56000	Madagascar, Petriky	FJ409926*	genseq-3
*Arthrosphaera brandtii* *	FMNH-INS 8650	Tanzania, Usambara Hills	FJ409915*	genseq-4
*Sphaeromimus musicus* 01*	FMNH-INS 56016	Madagascar, Andrahomana	FJ409919*	genseq-4
*Sphaeromimus musicus* 02*	FMNH-INS 56016	Madagascar, Andrahomana	FJ409920*	genseq-4
*Sphaeromimus musicus* 03*	FMNH-INS 56008	Madagascar, Mangatsiaka	FJ409921*	genseq-4
*Sphaeromimus musicus* 04*	FMNH-INS 56212	Madagascar, Tsimelahy	FJ409922*	genseq-4
*Sphaeromimus musicus* 05 (red)	ZFMK MYR 2273	Madagascar, Tsimelahy	KJ13244	genseq-4
*Sphaeromimus musicus* 06 (red)	ZFMK MYR 2276	Madagascar, Tsimelahy	KJ13245	genseq-4
*Sphaeromimus splendidus* A*	FMNH-INS 6702	Madagascar, Sainte Luce S9	FJ409918*	genseq-3
*Sphaeromimus splendidus* B*	FMNH-INS 56031	Madagascar, Sainte Luce S9	FJ409917*	genseq-3
*Sphaeromimus inexpectatus* A*	FMNH-INS 56033	Madagascar, Enato	FJ409916*	genseq-4
*Sphaeromimus inexpectatus* B	FMNH-INS 61090	Madagascar, Enato	KJ13246	genseq-4
*Sphaeromimus titanus* sp. n.	CASENT 9032789	Madagascar, Manombo,	KJ13247	genseq-1
*Sphaeromimus ivohibe* sp. n.	FMNH-INS 8184	Madagascar, Ivohibe	KJ13248	genseq-1
*Sphaeromimus lavasoa* sp. n. A*	FMNH-INS 56208	Madagascar, Gr. Lavasoa	FJ409924*	genseq-2
*Sphaeromimus lavasoa* sp. n. B	FMNH-INS 61143	Madagascar, Gr. Lavasoa	KJ13249	genseq-2
*Sphaeromimus lavasoa* sp. n. C	FMNH-INS 61142	Madagascar, Gr. Lavasoa	KJ13250	genseq-2
*Sphaeromimus andohahela* sp. n. 01	FMNH-INS 61135	Madagascar, Isaka-Ivondro	KJ13251	genseq-2
*Sphaeromimus andohahela* sp. n. 02	FMNH-INS 61137	Madagascar, Isaka-Ivondro	KJ13252	genseq-2
*Sphaeromimus andohahela* sp. n. 03	ZFMK MYR 2322	Madagascar, Isaka-Ivondro	KJ13253	genseq-1
*Sphaeromimus andohahela* sp. n. 04	FMNH-INS 61136	Madagascar, Isaka-Ivondro	KJ13254	genseq-2
*Sphaeromimus andohahela* sp. n. 05	FMNH-INS 61132	Madagascar, Manantantely	KJ13255	genseq-4
*Sphaeromimus andohahela* sp. n. 06	FMNH-INS 61138	Madagascar, Manantantely	KJ13256	genseq-4
*Sphaeromimus andohahela* sp. n. 07*	FMNH-INS 56210	Madagascar, Malio	FJ409923*	genseq-4
*Sphaeromimus andohahela* sp. n. 08	FMNH-INS 56210	Madagascar, Malio	KJ13257	genseq-4
*Sphaeromimus andohahela* sp. n. 09	ZFMK MYR 2323	Madagascar, Malio	KJ13258	genseq-4
*Sphaeromimus saintelucei* sp. n.	ZFMK MYR 889	Madagascar, Sainte Luce S8	KJ13259	genseq-1
*Sphaeromimus andrahomana* sp. n. Cave*	FMNH-INS 56211	Madagascar, Andrahomana	FJ409924*	genseq-4
*Sphaeromimus andrahomana* sp. n.	FMNH-INS 56214	Madagascar, Ankapaky Plateau	KJ13260	genseq-1
*Sphaeromimus* sp. ‚Vevembe‘	CASENT 9032816	Madagascar, Vevembe	KJ13261	genseq-4

### DNA analysis

To find the best substitution model, modeltest implemented in MEGA 5.05 (Tamura et al. 2011) was utilized. Codon positions included were 1st+2nd+3rd+Noncoding. All positions containing gaps and missing data were eliminated. There were a total of 567 positions in the final dataset. The lowest Bayesian Information Criterion score of 8149.1 was obtained by the GTR model plus invariant sites and gamma distribution to be best fitting (FreqA = 0.2694, FreqC = 0.2286, FreqT = 0.3304, FreqG = 0.1716, Invariant sites = 0.535, gamma shape = 1.29762). Maximum likelihood analyses were conducted in MEGA5 (Tamura et al. 2011). The bootstrap consensus tree (Fig. 20) from 1000 replicates (Felsenstein 1985) is taken to represent the evolutionary history of the analyzed taxa. All positions containing gaps and missing data were eliminated. There were a total of 570 positions in the final dataset. Mean uncorrected pairwise distances between terminals (transformed into percentages) were determined using MEGA5 (Tamura et al. 2011).

### Museum acronyms

CAS California Academy of Sciences, San Francisco, California, U.S.A.

FMNH Field Museum, Chicago, Illinois, U.S.A.

MNHN Muséum national d’Histoire naturelle, Paris, France.

QVMAG Queen Victoria Museum and Art Gallery, Launceston, Australia.

ZFMK Zoologisches Forschungsmuseum A. Koenig, Bonn, Germany.

## Results

### Genus Sphaeromimus

de Saussure & Zehntner, 1902

http://species-id.net/wiki/Sphaeromimus

Sphaeromimus de Saussure & Zehntner, 1902: 20 (first description); Attems 1926: 119 (list); Attems 1943: 60 (list); Jeekel 1971: 28 (list); Jeekel 1974: 45 (classification); Hoffman 1980: 63 (list); Jeekel 1999: 8 (catalogue, discussion); Enghoff 2003: 618 (list); Wesener and Sierwald 2005: 557 (redescription, additional species); Wesener and Wägele 2007: 147 (ecology); Wesener 2009: 8 (key); Wesener and VandenSpiegel 2009: 548 (morphological phylogenetic analysis); Wesener et al. 2010: 1185 (molecular phylogenetic analysis); Wesener 2014b: (morphological phylogenetic analysis).

#### Type species.

*Sphaeropoeus musicus* de Saussure & Zehntner, 1897, by monotypy.

#### Other species included (9).

*Sphaeromimus splendidus* Wesener & Sierwald, 2005

*Sphaeromimus inexpectatus* Wesener & Sierwald, 2005

*Sphaeromimus titanus* sp. n. Wesener

*Sphaeromimus vatovavy* sp. n. Wesener

*Sphaeromimus lavasoa* sp. n. Wesener

*Sphaeromimus andohahela* sp. n. Wesener

*Sphaeromimus ivohibe* sp. n. Wesener

*Sphaeromimus saintelucei* sp. n. Wesener

*Sphaeromimus andrahomana* sp. n. Wesener

#### Remarks.

See Wesener and Sierwald (2005) for a detailed revision of the genus. A phylogeny, highlighting the close relationship of *Sphaeromimus* to the Indian *Arthrosphaera* Pocock, 1895 is available based on morphological (Wesener and VandenSpiegel 2009; Wesener 2014b), and molecular characters (Wesener et al. 2010). A short re-diagnosis is presented so that fewer characters need to be mentioned in the species descriptions.

#### New diagnosis.

**Figure 1.**
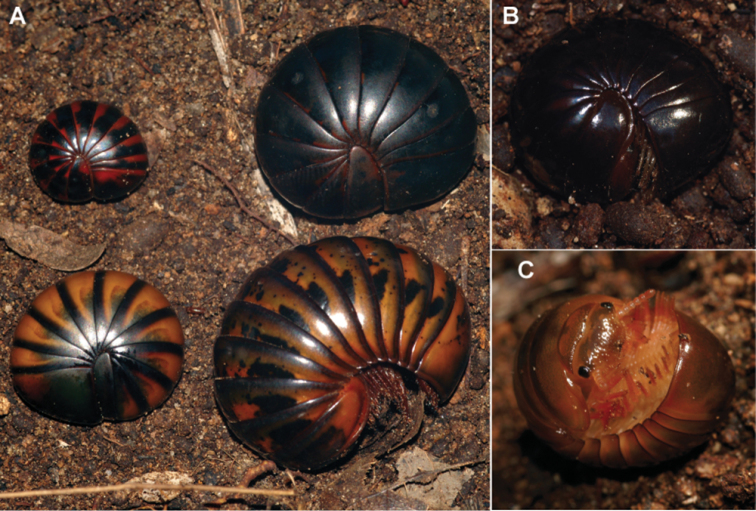
Photographs of living *Sphaeromimus*. **A** *Sphaeromimus musicus* (de Saussure & Zehntner, 1897) from Tsimelahy, upper left to lower left: red colour morph, black colour morph, normal colour morph, the similar looking sympatric *Zoosphaerium blandum* (de Saussure & Zehntner, 1902) **B** *Sphaeromimus lavasoa* sp. n. **C** *Sphaeromimus andrahomana* sp. n. cave specimen. Not to scale.

Genus of small to medium-sized (15–45 mm length) Arthrosphaeridae. Colour variable, rarely pink or with a reddish-black pattern (Fig. 1A), but usually black to brown (Fig. 1B). Head with short antennae consisting of well-rounded antennomeres lacking cuticular scales. Antennomere 6 massive, only antennomere carrying sensilla basiconica. Apical disc with numerous apical cones (≫20), number of cones sexual dimorphic, males with twice or even three times as many cones as females. Eyes consisting of 55–95 ocelli. Mandible with six or seven pectinate lamellae and a 3-combed internal tooth. Gnathochilarium typical of the order, rudimentary lateral palpi carrying three or four sensory cones. Tergites with a smooth surface, in some species polished. Legs short and broad, tarsus usually 2.5–4 times longer than wide. Leg pair 1 and 2 lacking an apical spine. Femur often with a well-developed toothed ridge. Coxae with a more or less well-developed sharp process carrying small triangular spines. Anal shield usually well-rounded, underside carrying a single, short, black locking carina located closely to the margin.

Male gonopore typical of Arthrosphaeridae, covered by a simple sclerotized plate.

Anterior telopod consisting of syncoxite plus four telopoditomeres. First telopoditomere enlarged, carrying the male stridulation organ (harp) consisting of 3–6 stridulation ribs, number of ribs independent of the size of the male, species specific. Second telopoditomere posteriorly with a process protruding up to the start of telopoditomere 4. Third telopoditomere short, sometimes with a spine juxtaposed to process of telopoditomere 2. Telopoditomere 4 larger than 3 and 4 combined, conical, basally with a field of sclerotized spots juxtaposed to process of telopoditomere 2, apically with a large, triangular lobe-like spine, usually three smaller spines distributed across the joint.

Posterior telopods always consisting of syncoxite with inner horns and inner lobes and pair of telopodites each consisting of three telopoditomeres forming chelae. First telopoditomere unremarkable, second telopoditomere forming the immovable finger of the chela. Immovable finger basally wide, apically tapering, always curved toward telopoditomere 3 (movable finger). Immovable finger as long as, or in some species overlapping movable finger. Membrane of telopoditomere 2 often extended into lobe, an anterior side juxtaposed to telopoditomere 3 with a dense field of sclerotized spots. Telopoditomere 3 curved or straight, relatively slender, margin juxtaposed to immovable finger with 20–40 small black crenulated teeth, three or four spines, and one, rarely two (*Sphaeromimus ivohibe* sp. n.) large membranous lobes.

Female vulva atypical for Arthrosphaeridae, with external and inner plate standing below operculum, not extending mesally. Operculum large and well-rounded, protruding at least up to basal half of prefemur. Female subanal plate greatly enlarged, in some species almost divided into two plates. Subanal plate carrying a washboard with 3–8 stridulation ribs on each side, number of ribs depending on female body size.

#### Key to species of *Sphaeromimus*

**Table:** 

1	Male harp on the anterior telopod with 3 stridulation ribs (Fig. 2E)	2
–	Male harp on the anterior telopod with 4–6 stridulation ribs (Fig. 9A)	7
2	Body length <20 mm. Midbody legs without a coxal lobe (Fig. 17A). Endotergum with single row of marginal bristles (Fig. 16B). Immovable finger of posterior telopod slender, apically strongly curved like a hook (Fig. 17F). Colour pink or brown	3
–	Body length >20 mm. Midbody legs at least with weak coxal lobe (Fig. 2C). Endotergum usually with at least two (Fig. 12A), rarely only one (*Sphaeromimus titanus* sp. n.) row of marginal bristles. Immovable finger of posterior telopod at least basally wide (Fig. 3B). Colour black or brown	4
3	Colour pink, surface shiny. Process of telopoditomere 2 of anterior telopods in anterior view visible laterally. Littoral forest of Mandena and rainforest of Enato	*Sphaeromimus inexpectatus*
–	Colour light brown, surface dull. Process of telopoditomere 2 of anterior telopods in anterior view not visible (Fig. 17B). Littoral forest of Sainte Luce, fragment S8	*Sphaeromimus saintelucei* sp. n.
4	Body length >30 mm, light brown. Endotergum with single row of marginal bristles (Fig. 5A). Movable finger of posterior telopod straight (Fig. 3B). Lowland forest of Manombo	*Sphaeromimus titanus* sp. n.
–	Body length 21–28 mm, black or dark brown. Endotergum with two rows of marginal bristles (Fig. 12A). Movable finger of posterior telopod curved (Fig. 11D)	5
5	Midbody legs with strongly developed coxal process. Tergite surface shiny. Littoral forest of Sainte Luce, fragment S9	*Sphaeromimus splendidus*
–	Midbody legs with barely developed coxal process (Fig. 11A). Tergite surface dull	6
6	Endotergum with strongly developed cuticular patterns (Fig. 12A). Movable finger of posterior telopod without small pits, carrying 20–22 crenulated teeth (Fig. 14D). Andohahela mountain chain, Manantantely, Malio and Isaka-Ivondro	*Sphaeromimus andohahela* sp. n.
–	Endotergum with weakly developed cuticular patterns (Fig. 16C). Movable finger of posterior telopod covered with small pits, carrying 23 or 24 crenulated teeth (Fig. 18F). Inside Grotte d’Andrahomana and in deep ravines N of Ankapaky	*Sphaeromimus andrahomana* sp. n.
7	Unique black pattern on orange-reddish basic colour (Fig. 1A). Harp with 5 ribs. Endotergum with three rows of marginal bristles. Widespread in the SW spiny forest	*Sphaeromimus musicus*
–	Colour different, either uniformly black or brown (Fig. 1B). Rainforest species	8
8	Harp with 4 stridulation ribs (Fig. 11B). Endotergum with two rows of marginal bristles, not reaching tergite margin (Fig. 12A). Operculum well-rounded (Fig. 11F). Lavasoa Mountain	*Sphaeromimus lavasoa* sp. n.
–	Harp with 5 or 6 stridulation ribs (Fig. 9A). Endotergum with single row of marginal bristles (Fig. 5B)	9
9	Harp with 5 stridulation ribs (Fig. 9A). Marginal bristles of endotergum protruding beyond tergite margin (Fig. 5B). Operculum apically recessed (Fig. 9F). Movable finger of posterior telopod with single membranous lobe (Fig. 9E). Vevembe-Vatovavy area	*Sphaeromimus vatovavy* sp. n.
–	Harp with 6 stridulation ribs (Fig. 15D). Marginal bristles sparse and very short (Fig. 16A). Movable finger of posterior telopod with two membranous lobes (Fig. 15H). Small, black species, red legs. Ivohibe mountain	*Sphaeromimus ivohibe* sp. n.

### Sphaeromimus musicus

(de Saussure & Zehntner, 1897)

http://species-id.net/wiki/Sphaeromimus_musicus

Figure 1A

Sphaeropoeus musicus de Saussure & Zehntner, 1897: pl. 4, fig. 1 a-e (first description)
Sphaeromimus musicus : de Saussure and Zehntner 1902: 75 (description); Jeekel 1999: 8 (list); Enghoff 2003: 618 (list); Wesener and Sierwald 2005: 564 (redescription); Wesener 2009: 131 (list); Wesener and VandenSpiegel 2009: 548 (morphological phylogenetic analysis); Wesener et al. 2010: 1185 (molecular phylogenetic analysis); Wesener 2014b: (morphological phylogenetic analysis).

#### Additional specimen records.

5 ♂ & ♀, ZFMK MYR2273, Madagascar, Province Toliara, PN Andohahela, Tsimelahy, 24°57.296'S, 046°37.214'E, 135 m, spiny forest, close to river, coll. Wesener & Schütte, 24.v.2007; 2 ♂, ZFMK MYR2276, PN Andohahela, Tsimelahy, 24°57.296'S, 046°37.214'E, 135 m, spiny forest, close to river, coll. Wesener & Schütte, 24.v.2007; 5 ♂ & ♀, FMNH-INS 56027, same data as previous; 7 ♂ & ♀, ZFMK MYR2274, Grotte Andrahomana, 24°51.006'S, 046°55.907'E, dry forest plateau, coll. Wesener & Schütte, 20.v.2007; 4 ♂ & ♀, FMNH-INS 56016, same data as previous; 6 ♂ & ♀, ZFMK MYR2279, PN Andohahela, Mangatsiaka, 24°58.051'S, 046°33.206'E, 90 m, spiny forest, coll. Wesener & Schütte, 23.v.2007; 5 ♂ & ♀, FMNH-INS 56008, same data as previous; 6 ♂ & ♀, FMNH-INS 7822, Province Antananarivo, Forêt de Analavelona, Antanimena, 12.5 km NW Andramoheza, 22.6783°S, 44.1917°E, 1050 m, coll. S. M. Goodman, 9–15.3.1998; 1 ♀, MNHN TW29, Madagascar, Province Toliara, Entree nº1, 1927, envoi G. Petit, Madagascar, Caisse 7 (tuite de lemoir la caisse 6), Petit, 1926, Bords du Fiherenana, P de Tulear, 3°Envoi, most likely “Bords du Fiherenana”, 23.01°S, 44.09°E; 1 ♂, MNHN TW120, Madagascar, Mission R. Decary, Fort Dauphin, Juin 1926, entree nº17, 1927.

Localities. S. musicus is apparently widespread in the southern spiny forest ecosystem. Different colour morphs from the same locality (Fig. 1A) showed identical COI sequences (Fig. 20), while the intraspecific variation between two populations 35 km apart was 2%.

### Sphaeromimus splendidus

Wesener & Sierwald, 2005

http://species-id.net/wiki/Sphaeromimus_splendidus

Sphaeromimus splendidus Wesener & Sierwald, 2005: 567 (first description); Wesener and Wägele 2007: 150 (ecology); Wesener 2009: 131 (list); Wesener and VandenSpiegel 2009: 548 (morphological phylogenetic analysis); Wesener et al. 2010: 1185 (molecular phylogenetic analysis); Wesener 2014b: (morphological phylogenetic analysis).

#### Additional specimen records.

1 ♀ paratype, **ZFMK MYR2272** (tranferred from FMNH), coll. T. Wesener, 06.iv.2003; 11 ♂ & ♀, **ZFMK MYR2271**, coll. Wesener & Schütte, 01.vi.2007; 3 ♂ & ♀, **ZFMK MYR2277**, same data as previous; 19 ♂ & ♀, **FMNH-INS 56031**, same data as previous.

#### Localities.

Only recorded from the littoral rainforest at Sainte Luce, fragment S9 (Wesener and Sierwald 2005). The two specimens from which the COI gene was sequenced differ by a single base pair substitution.

### Sphaeromimus inexpectatus

Wesener & Sierwald, 2005

http://species-id.net/wiki/Sphaeromimus_inexpectatus

Sphaeromimus inexpectatus Wesener & Sierwald, 2005: 570 (first description); Wesener and Wägele 2007: 150 (ecology); Wesener 2009: 131 (list); Wesener and VandenSpiegel 2009: 548 (morphological phylogenetic analysis); Wesener et al. 2010: 1185 (molecular phylogenetic analysis); Wesener 2014b: (morphological phylogenetic analysis).

#### Additional specimen records.

3 ♂ & ♀, ZFMK MYR2275, Enato, 24°53'0.25"S, 046°59'2.77"E, rainforest, coll. Wesener & Schütte, 27.v.2007; 7 ♂ & ♀, ZFMK MYR2278, same data as previous; 1 ♂, FMNH-INS 61090, same data as previous; 1 ♂, FMNH-INS 61091, same data as previous; 8 ♂ & ♀, FMNH-INS 56033, same data as previous.

#### Localities.

Only recorded from the littoral rainforest of Mandena (Wesener and Sierwald 2005) and the rainforest of Enato (Wesener and Wägele 2007). The two specimens from which the COI gene was sequenced, both from Enato, differ in four base pair substitutions.

### Sphaeromimus titanus

Wesener sp. n.

http://zoobank.org/A178FBE5-A0FD-43BF-B244-5FF116D8C720

http://species-id.net/wiki/Sphaeromimus_titanus

Figures 2 – 4 , 5A

#### Material examined.

Type material. *Holotype*: 1 ♂, BLF13962 (CASENT 9032789), Madagascar, Province Fianarantsoa, Réserve Speciale Manombo, 24.5 km 228°SW Farafangana, 23°00'57"S, 047°43'08"E, 30 m, rainforest, coll. Brian L. Fisher et al., 20.iv.2006, general collecting.

**Paratype.** 1 ♀, same data as holotype.

#### Diagnosis.

By far the largest known *Sphaeromimus*, >30 mm. Differing from all other *Sphaeromimus* with three stridulation ribs on the male harp in the following characters: large size; first stigma-carrying plate with a well-rounded projecting apex; tarsus with few hairs, relatively slender, 4.5 times longer than wide; endotergum with single regular row of long setae that barely protrude up to posterior margin; chela of posterior telopod in posterior view almost glabrous, movable finger straight.

#### Description.

**Figure 2.**
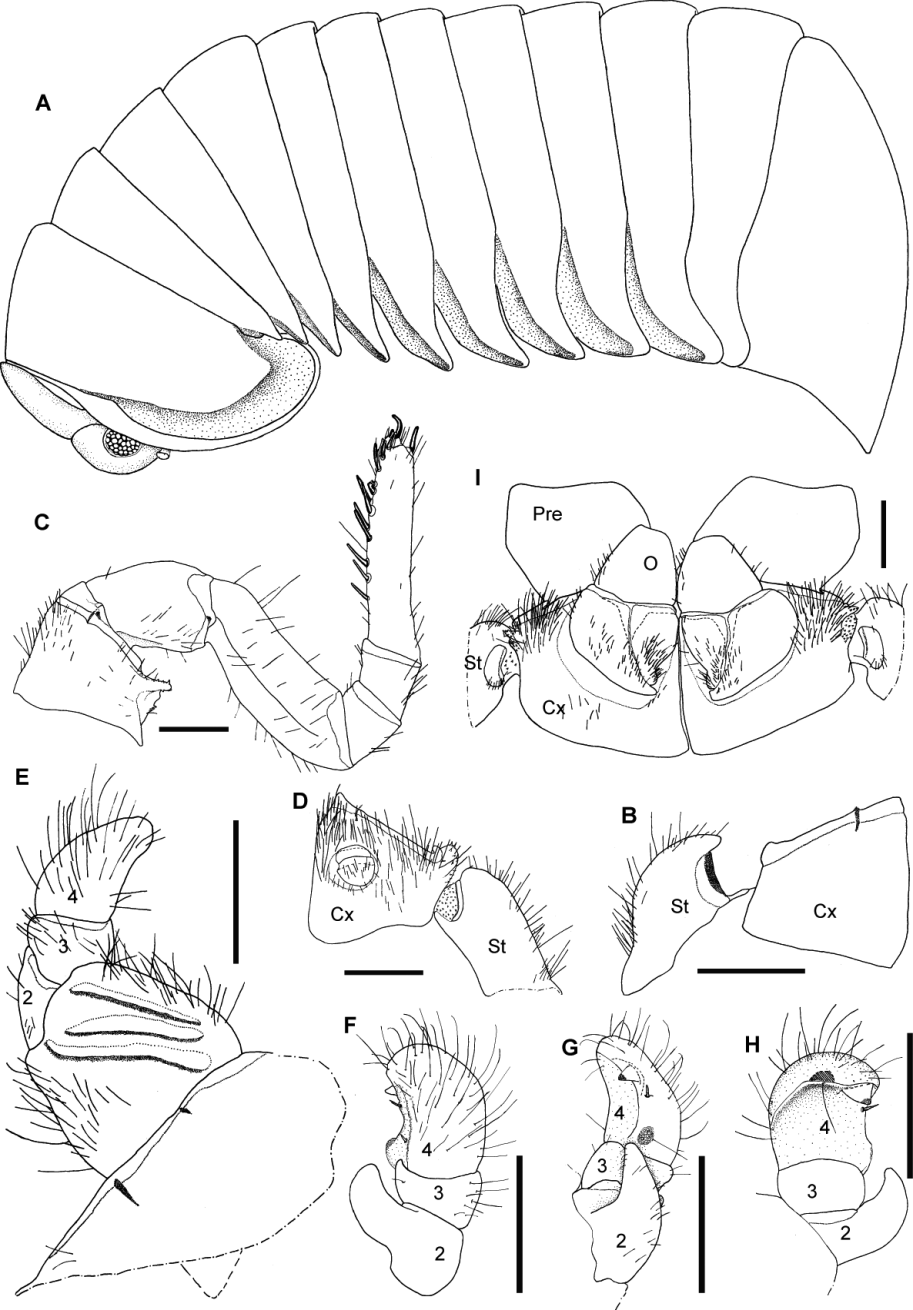
*Sphaeromimus titanus* sp. n., **A, I** female paratype **B–H** holotype **A** habitus **B** first right stigmatic plate and coxa 1 **C:** left leg 9 **D** left coxa 2 with gonopore and stigmatic plate 2 **E** left anterior telopod, anterior view ♀ left anterior telopod, mesal view **G** left anterior telopod, posterior view **H** left anterior telopod, lateral view **I** coxae and prefemora 2 with vulvae. Abbreviations: Cx = coxa; O = operculum; Pre = prefemur; St = stigmatic plate. Scale bars = 1 mm.

**Measurements.** Male holotype: 33.5 long, 16.6 (2nd), 17.2 (8th) wide, 9.2 (2nd), 11.1 (9th - highest) height. Female: 46.9 mm long, 24.15 mm (2nd), 27.4 (8th - widest) wide, 13.1 (2nd), 19.5 mm (12th, highest) high (Fig. 2A).

Colouration of tergites dark brown with black posterior margin. Paratergite impressions and groove of thoracic shield orange. Legs, antennae and pleurites orange, head and collum dark brownish-black, eyes green.

Head: Eyes with >70 ocelli. Antennae short, protruding back to coxa 6. Antennomeres 1–4 with few longer setae, 5 and 6 densely pubescent (Fig. 4A). Antennomere 6 towards disc with single row of sensilla basiconica (Fig. 4A). Female with 36/45, male with 79/81 apical cones (Fig. 4B). Mouthparts not dissected.

Collum glabrous except few setae at margins.

Thoracic shield smooth and glabrous, few setae in grooves. Grooves deep (Fig. 2A). Tergites 3–12 smooth, paratergite tips of midbody tergites strongly projecting posteriorly (Fig. 2A).

Anal shield massive, with a steep edge (Fig. 2A), lacking pubescent area.

Endotergum inner section with numerous short triangular spines and long setae (Fig. 5A). Between ridge and inner area two rows of weakly impressed, circular cuticular impressions. Externally single, sparse row of marginal bristles (Fig. 5A). Bristles short, barely reaching to tergite margin.

First stigma-carrying plate with a well-rounded projecting apex (Fig. 2B).

Leg 1 with 2 or 3, 2 with 4–6, 3 with 10 ventral spines. Leg pairs 4–21 with 11–14 ventral spines. Coxa process strongly developed (Fig. 2C). Femur 2.1, tarsus 4.4 times longer than wide. All podomeres with only few setae (Fig. 2C).

Male gonopore typical for the genus (Fig. 2D).

Anterior telopod (Fig. 2E–H): Harp carrying three stridulation ribs (Fig. 2E). Shape usual for the genus, telopoditomere 4 with one large triangular spine and 2 or 3 smaller ones (Fig. 2F–H), apically with a weakly sclerotized spot (Fig. 2H).

**Figure 3.**
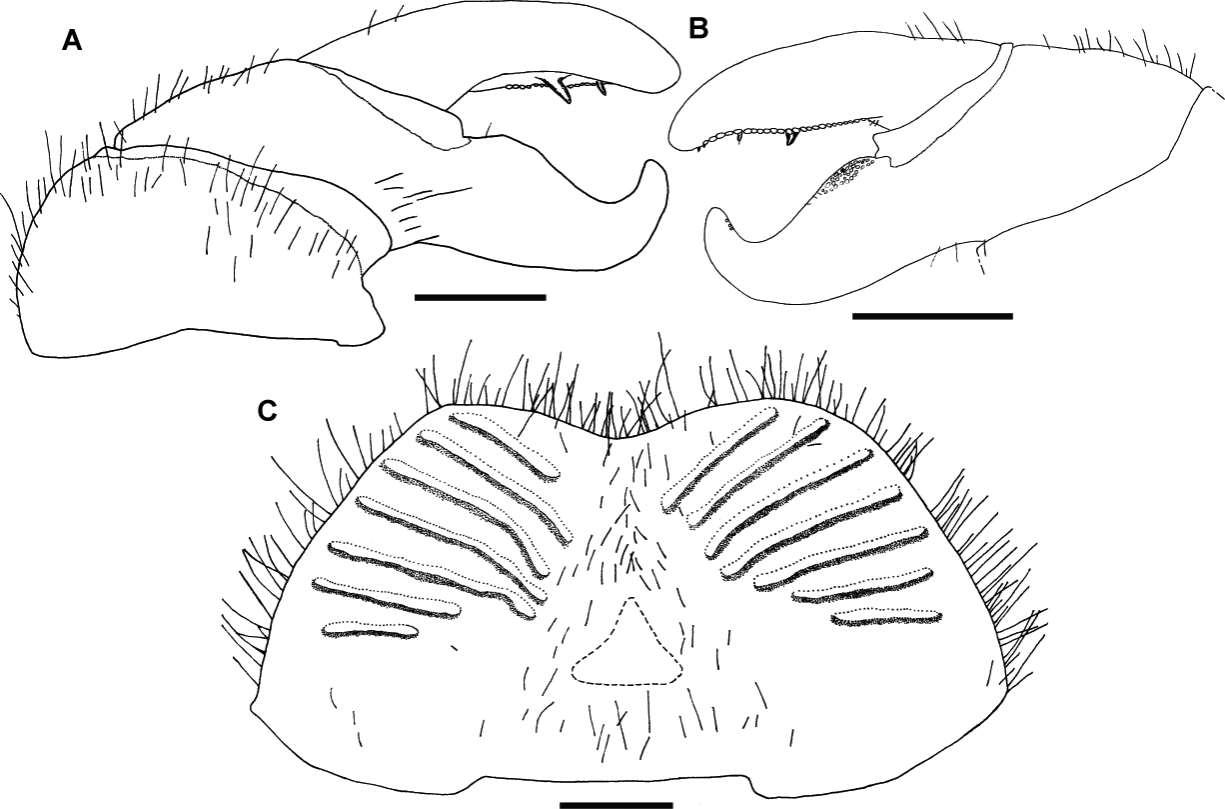
*Sphaeromimus titanus* sp. n., **C** female paratype, **A, B** holotype **A** left posterior telopod, anterior view **B** left posterior telopod, posterior view **C** female subanal plate with washboard. Scale bars = 1 mm.

Posterior telopod (Fig. 3A, B): Podomere 3 straight, 3.3 times longer than wide, slightly longer than immovable finger (Fig. 3A). Hollowed-out inner margin with one lobe and three sclerotized spines, posterior aspect with *ca.* 24 small crenulated teeth. Immovable finger basally wide, apically tapering, weakly curved towards podomere 3. Podomere 1 with few setae (Fig. 3A), podomere 2 only with few setae at anterior side, posterior side glabrous (Fig. 3B). Podomere 3 with only few marginal setae.

**Figure 4.**
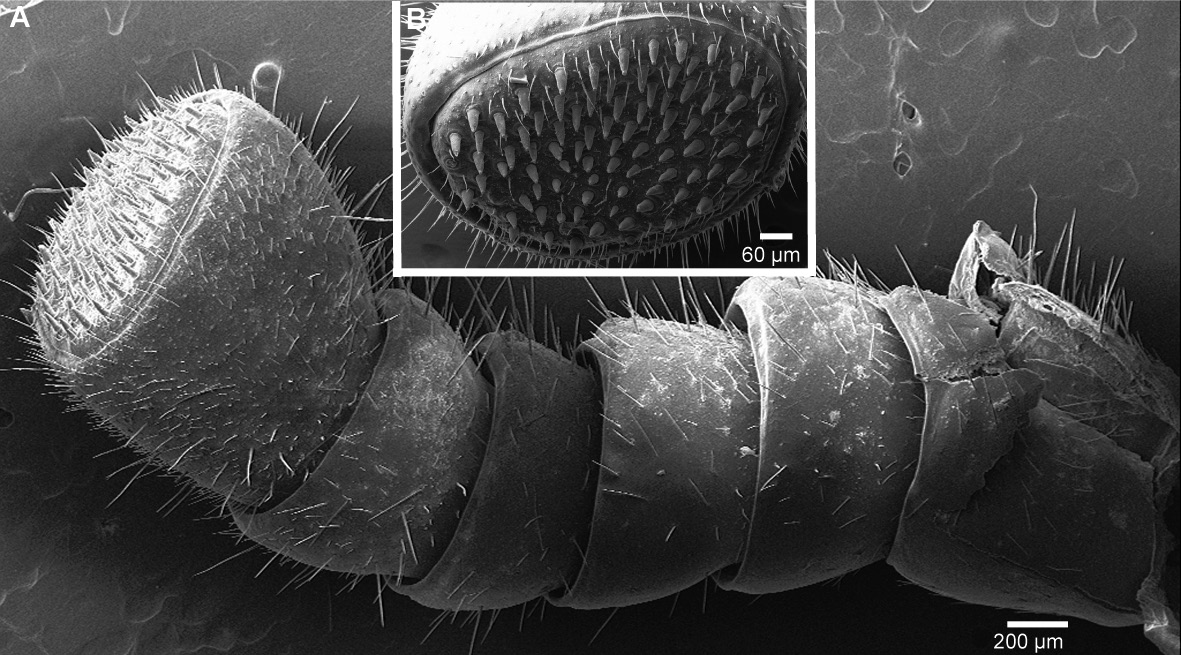
*Sphaeromimus titanus* sp. n., holotype, SEM, left antenna. **A** lateral view **B** detail of disc with apical cones.

**Figure 5.**
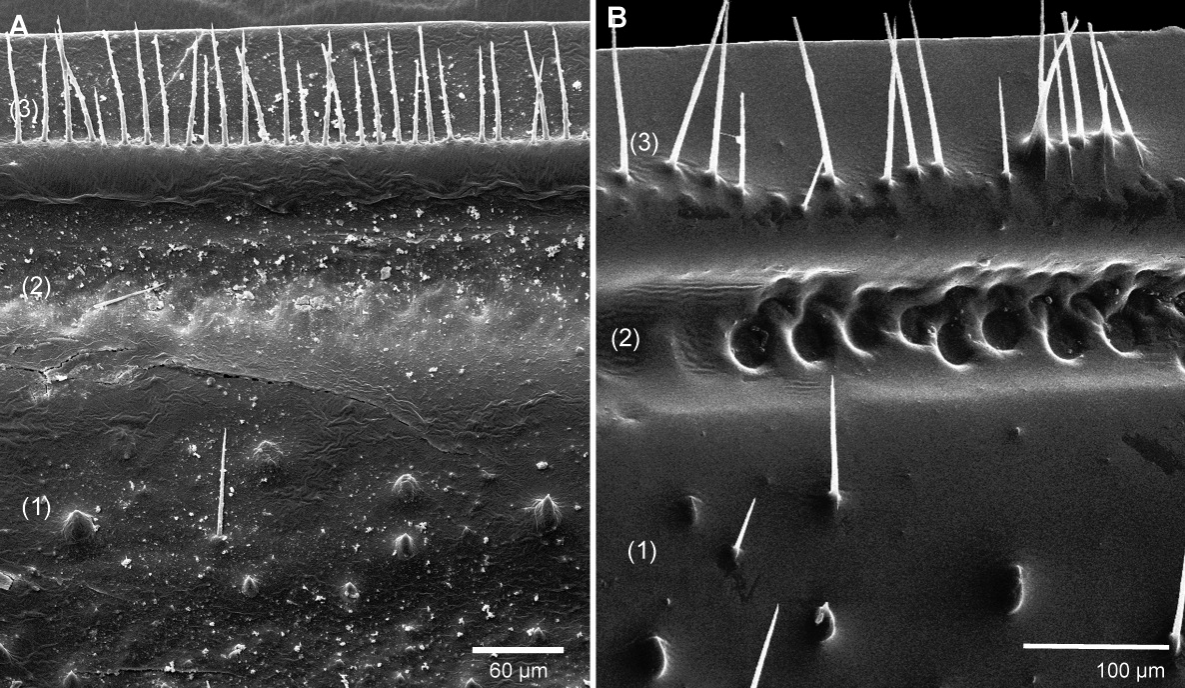
SEM, Endoterga of mid-body tergite. **A** *Sphaeromimus titanus* sp. n., holotype **B** *Sphaeromimus vatovavy* sp. n., holotype. Abbreviations: (1) = inner area with large spines and long setae; (2) = area with cuticular patterns; (3) = outer area with row(s) of marginal bristles and tergite margin.

Female sexual characters: Vulva massive. Operculum well-rounded, protruding up to basal half of prefemur (Fig. 2I). Subanal shield massive, with shallow invagination at apical margin. Washboard with seven stridulation ribs on each side (Fig. 3C).

#### Etymology.

‘titanus’, adjective, referring to the large size of the species.

#### Distribution.

Only known from the eastern lowland rainforest of Manombo, which is now isolated by vast areas of pseudosteppe from all other remaining rainforests.

### Sphaeromimus vatovavy

Wesener sp. n.

http://zoobank.org/19F8614B-296B-44A0-A203-F1C38CE54FD3

http://species-id.net/wiki/Sphaeromimus_vatovavy

**Figure 6.**
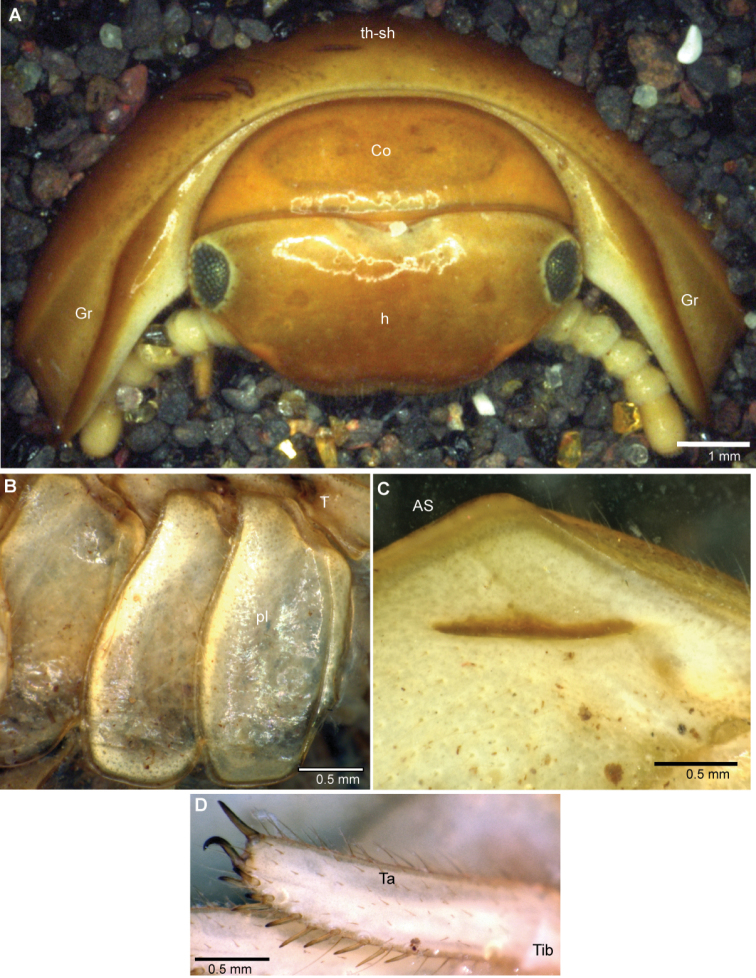
*Sphaeromimus vatovavy* sp. n., female paratype, multi-layer photographs. **A** head with collum and thoracic shield, frontal view **B** pleurites **C** underside of anal shield with black locking carina **D** tarsus of midbody leg. Abbreviations: AS = anal shield; Co = collum (tergite 1); Gr = lateral grooves of thoracic shield; h = head; pl = pleurite; T = tergite; Ta = tarsus; th-sh = thoracic shield (tergite 2); Tib = tibia.

**Figure 7.**
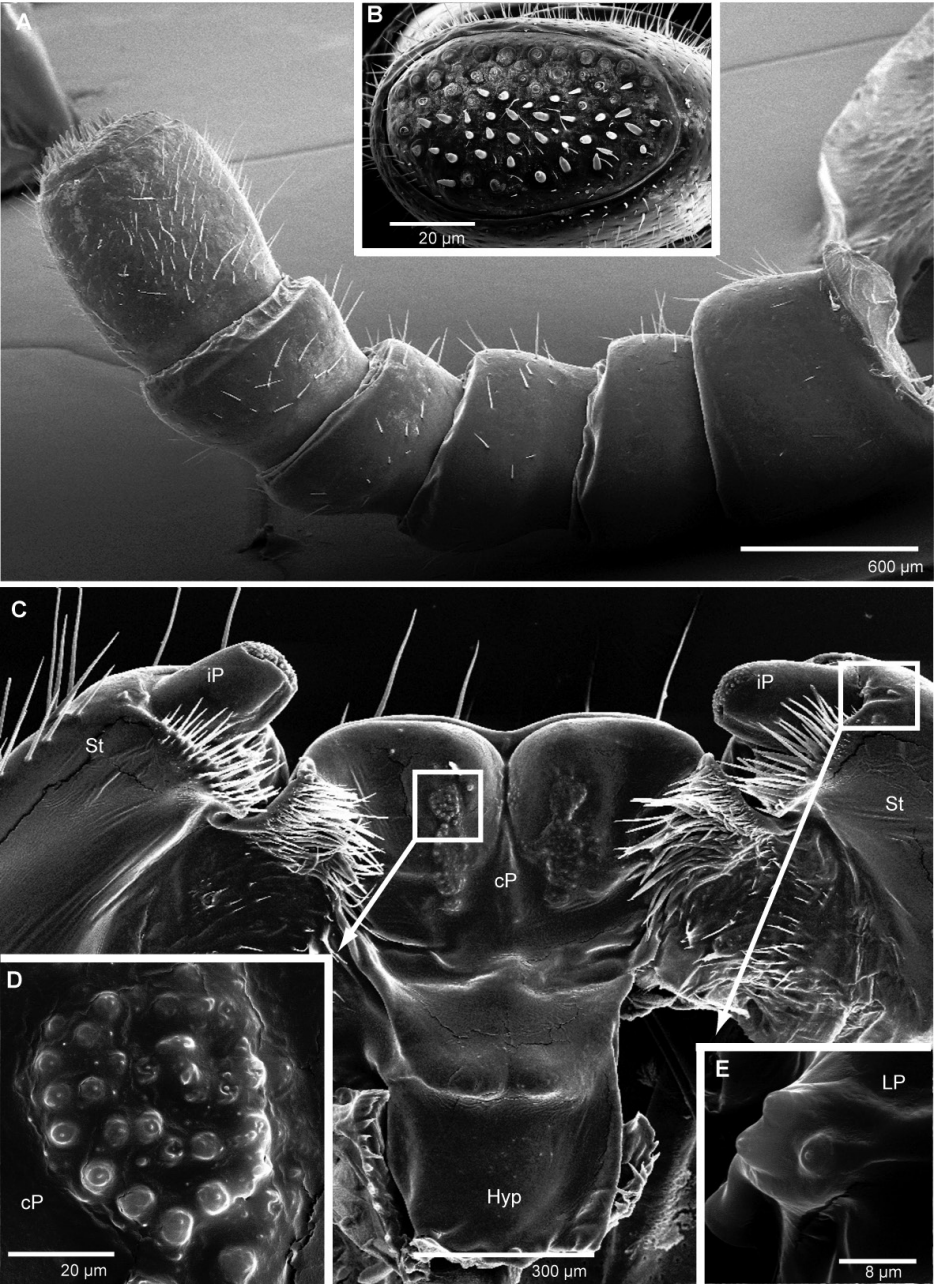
*Sphaeromimus vatovavy* sp. n., female paratype, SEM. **A** right antenna, lateral view **B** antennomere 6 with disc **C** gnathochilarium, underside **D** detail of sensory cones on central pad **E** rudimentary right lateral palpus. Abbreviations: cP = central pads; Hyp = hypopharyngeal area; iP = inner palpus; LP = rudimentary lateral palpus; St = stipites.

**Figure 8.**
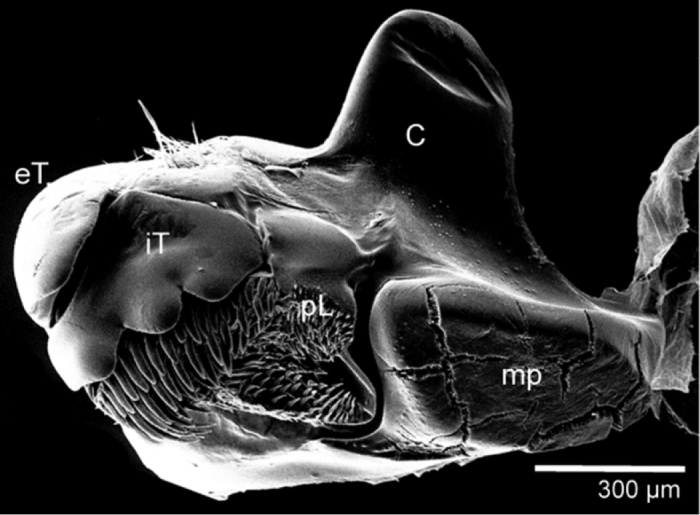
*Sphaeromimus vatovavy* sp. n., female paratype, SEM. Right mandible. Abbreviations: C = condylus; eT = external tooth; iT = combed inner tooth; mp = molar plate; pL = pectinate lamellae.

**Figure 9.**
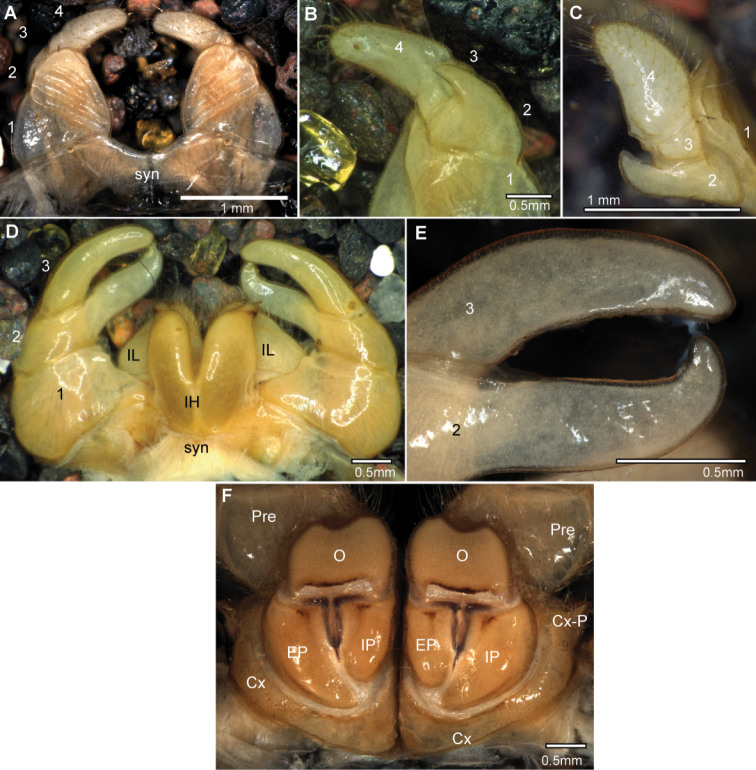
*Sphaeromimus vatovavy* sp. n., A–E male holotype, ♀ female paratype, multi-layer photographs. **A** anterior telopods, anterior view **B** left anterior telopod, posterior view **C** left anterior telopod, lateral view **D** posterior telopods, anterior view **E** chela of right posterior telopod, posterior view ♀ coxa and prefemur 2 with vulvae. Abbreviations: Cx = coxa; Cx-P = coxal process; EP = external, lateral plate of vulva; IH = inner horns; IL = inner lobes; IP = inner, mesal plate of vulva; O = operculum; Pre = prefemur; syn = syncoxite.

Figures 5B , 6 , 7 , 8 , 9

#### Material examined.

Type material. *Holotype*: 1 ♂, MNHN ‘39’, Madagascar, Province Fianarantsoa, Forêt primitive de Tsianovoha (=Vatovavy-Fitovinany, Fort Carnot), rainforest, coll. Mission Heim á Madagascar, 1934-35, fin Sept. 34.

**Paratype.** 1 ♀, same data as holotype.

#### Diagnosis.

Five stridulation ribs on the male harp, a character only shared with the spiny forest species *Sphaeromimus musicus*. Shape of female operculum unique, apically recessed. Endotergum with two rows of deeply impressed cuticular patterns and two dense, irregular rows of marginal bristles that protrude above tergite margin.

#### Description.

Measurements: Female paratype: 21.1 mm long, 10.2 mm wide (2nd), 5.8 mm height (2nd), male broken, not measured but slightly smaller.

Colouration influenced by 70 years in preservative, faded dark brown without any discernable pattern (Fig. 6A). Antennae, legs and pleurites faded olive green, eyes green (Fig. 6A–D).

Head: Eyes with >60 ocelli (Fig. 6A). Posterior margin of head towards collum glabrous (Fig. 6A). Antennae short, protruding laterally slightly past margins of thoracic shield (Fig. 6A). Antennomeres 1–5 with few longer setae, only antennomere 6 densely pubescent (Fig. 7A). Antennomere 6 towards disc with single row of sensilla basiconica (Fig. 7A). Female with 63/65 (Fig. 7B), male with >90 apical cones. Gnathochilarium typical for the order (Fig. 7C), central pads mainly with single type of sensilla (Fig. 7D), rudimentary lateral palpi consisting of three sensilla (Fig. 7E). Mandible with the typical shape of the order, inner tooth 3-combed, with six long pectinate lamellae, condylus with a sharp groove at its apex (Fig. 8).

Collum glabrous, with few setiferous points at its margin (Fig. 6A). Thoracic shield smooth and glabrous, except for margin and lateral grooves (Fig. 6A). Grooves deep, anterior brim swollen. Tergites 3–12 smooth, paratergite tips of midbody tergites slightly projecting posteriorly. Anal shield massive, well-rounded, with single locking carinae, as typical for *Sphaeromimus* (Fig. 6C).

Endotergum inner section with numerous short triangular spines and long setae (Fig. 5B). Between ridge and inner area two rows of strongly impressed, circular cuticular impressions. Externally two dense but irregular rows of marginal bristles (Fig. 5B). Bristles long, protruding beyond tergite margin.

First stigma-carrying plate with a well-rounded apex.

Leg 1 with 2–4, 2 with 3–5, 3 with 8 ventral spines. Leg pairs 4–21 with 10–12 ventral spines. Coxa process of midbody legs weakly developed. Femur 1.8, tarsus 3.3 times longer than wide (Fig. 6D).

Male gonopore inconspicuous.

Anterior telopod (Fig. 9A–C): Harp carrying five stridulation ribs (Fig. 9A). Shape usual for the genus, telopoditomere 4 massive, larger than two preceding joints, with one large triangular spine and 3 or 4 smaller ones (Fig. 9B, C).

Posterior telopod (Fig. 9D, E): Podomere 3 strongly curved, 3.1 times longer than wide, slightly longer than immovable finger (Fig. 9D). Hollowed-out inner margin with one lobe and four sclerotized spines, posterior aspect with *ca.* 26 small crenulated teeth (Fig. 9E). Immovable finger only slightly tapering apically, *ca.* 3 times longer than wide, apically strongly hooked towards podomere 3. Podomere 1 with few setae (Fig. 9D), podomere 2 and 3 glabrous.

Female sexual characters: Coxa process on leg 2 well-developed. Vulva massive. Operculum apically emarginate, protruding up to basal half of prefemur (Fig. 9F). Subanal partly reduced, with shallow invagination at apical margin. Washboard with 10 stridulation ribs on each side.

#### Etymology.

‘vatovavy’, noun in apposition, referring to the type locality, located in the area of Vatovavy-Fitovinany (Fort Carnot).

#### Distribution.

Only known from the eastern lowland rainforest of Tsianovoha. Satellite images do not show much remaining natural vegetation in the area.

### Sphaeromimus lavasoa

Wesener sp. n.

http://zoobank.org/8B359EBE-03AC-4367-96E7-D47F483447FE

http://species-id.net/wiki/Sphaeromimus_lavasoa

**Figure 10.**
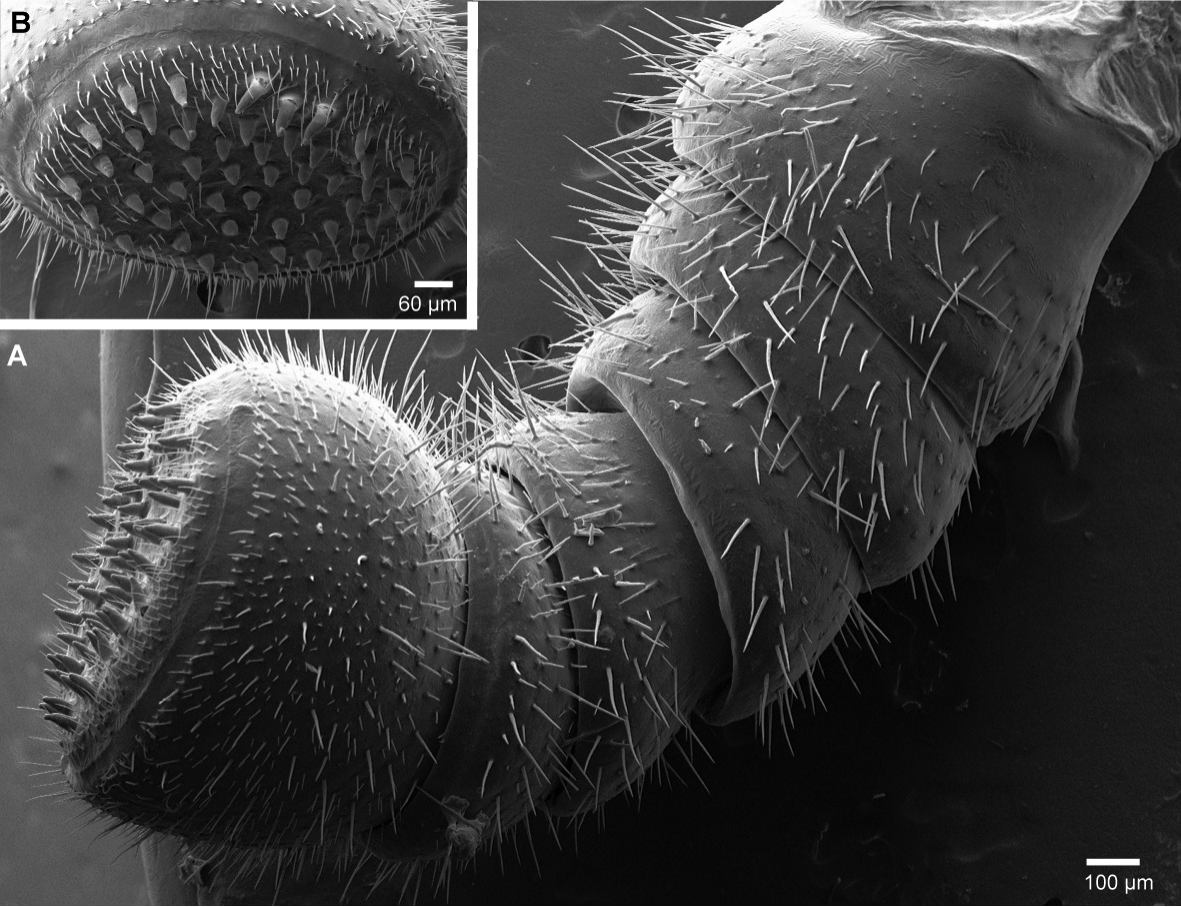
*Sphaeromimus lavasoa* sp. n., male paraype, SEM, left antenna. **A** lateral view **B** detail of disc with apical cones.

**Figure 11.**
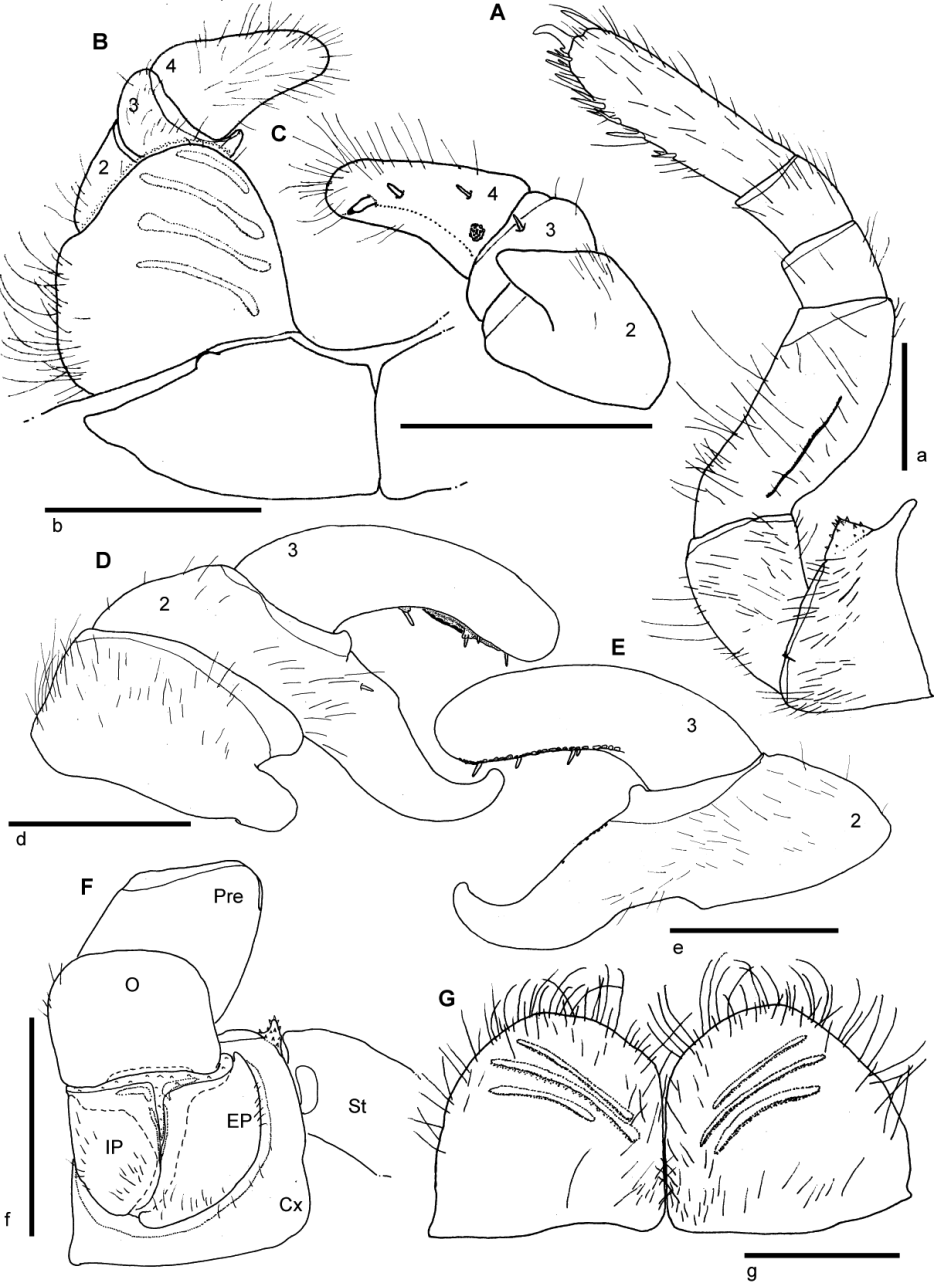
*Sphaeromimus lavasoa* sp. n., **A–E** male holotype ♀, **G** female paratype. **A** left leg 9 **B** anterior telopod, anterior view **C** left anterior telopod, posterior view **D** left posterior telopod, anterior view **E** chela of left posterior telopod, posterior view ♀ coxa and prefemur 2 with vulvae **G** female subanal plate with washboard. Abbreviations: Cx = coxa; EP = external, lateral plate of vulva; IP = inner, mesal plate of vulva; O = operculum; Pre = prefemur; St = stigmatic plate. Scale bars = 1 mm.

**Figure 12.**
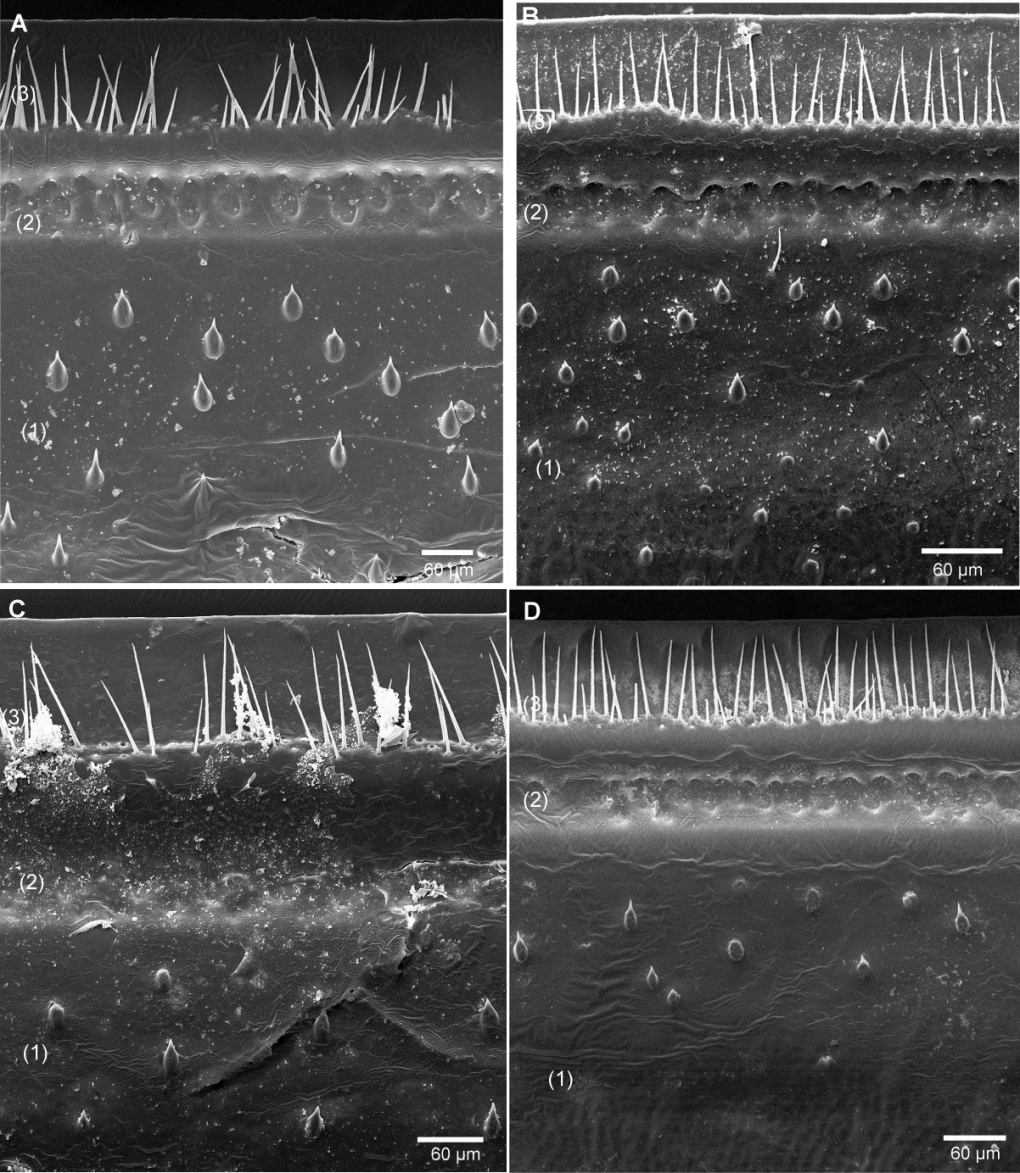
SEM, Endoterga of mid-body tergite. **A** *Sphaeromimus lavasoa* sp. n., paratype **B** *Sphaeromimus andohahela* sp. n., holotype from Isaka-Ivondro **C** specimen from Malio **D** specimen from Manantantely. Abbreviations: (1) = inner area with large spines and long setae; (2) = area with cuticular patterns; (3) = outer area with row(s) of marginal bristles and tergite margin.

Figs 1B , 10 , 11 , 12A

Sphaeromimus ‘sp. n. III G-Lavasoa’: Wesener et al. 2010: 1185 (molecular phylogenetic analysis).

#### Material examined.

Type material. *Holotype*. 1 ♂, ZFMK MYR2320, Madagascar, Province Toliara, Grande Lavasoa, 25°5'10.23"S, 46°44'55.93"E, 524 m, rainforest, coll. Wesener & Schütte, 14.vi.2007.

**Paratypes.** 2 ♀, ZFMK MYR2321, same data as holotype; 1 ♂, FMNH-INS 61141, same data as previous; 1 ♂, FMNH-INS 61134 (Antenna removed); 1 ♀, FMNH-INS 61142; 1 ♀, FMNH 61143; ~ 35 ♂, ♀, juv., FMNH-INS 56208, all same data as holotype; 1 ♀, FMNH-INS 56213, Petit Lavasoa, 25°05.021'S, 046°46.110'E, 668 m, rainforest, coll. Wesener & Schütte, 21.v.2007.

**Other material.** ~ 50 ♂, ♀, juv., same data as holotype, sent as voucher specimens to the University of Antananarivo.

#### Diagnosis.

Small shiny-black *Sphaeromimus* with brown collum and head. Male harp with four stridulation ribs. Midbody legs with weakly-developed coxal process.

#### Description.

Measurements: male holotype: 21.7 long, 10.4 (2nd), 11.2 (8th - widest) wide, 5.6 (2nd), 6.8 (10th - highest) height. Largest female (with eggs): 23.1 mm long, 11.4 mm (2nd), 12.1 (8th - widest) wide, 6.35 (2nd), 8.55 mm (10th, highest) high.

Colouration of tergites black. Collum and head light brown (Fig. 1B). Legs and antennae dark greenish-brown.

Head: Eyes with >60 ocelli. Antennae very short, protruding as far as leg 6. All antennomeres densely pubescent (Fig. 10A). Antennomere 6 towards disc with single row of sensilla basiconica (Fig. 10A). Female with 22/24 (largest), male with 62/65 apical cones (Fig. 10B). Mouthparts not dissected.

Collum glabrous except 3 or 4 short isolated setae on the surface and few at margin.

Thoracic shield smooth and glabrous, few setae in grooves. Tergites 3–12 smooth, paratergite tips of midbody tergites only weakly projecting posteriorly (Fig. 1B).

Anal shield massive, well-rounded, lacking pubescent area.

Endotergum inner section with numerous short triangular spines and very few setae (Fig. 12A). Between ridge and inner area two rows of weakly impressed, circular cuticular impressions. Externally two irregular, dense rows of marginal bristles (Fig. 12A). Bristles short, ending well before tergite margin.

First stigma-carrying plate with a well-rounded apex.

Leg 1 with 3 or 4, 2 with 5 or 6, 3 with 8 or 9 ventral spines. Leg pairs 4–21 with 11–13 ventral spines. Coxa process weakly developed (Fig. 11A). Femur 2, tarsus 3.1 times longer than wide.

Male gonopore inconspicuous.

Anterior telopod (Fig. 11B, C): Harp carrying four stridulation ribs (Fig. 11B). Shape usual for the genus, telopoditomere 4 with one large triangular spine and 2 smaller ones (Fig. 11C). Telopoditomere 3 with a spine juxtaposed to process of telopoditomere 2 (Fig. 11C).

Posterior telopod (Fig. 11D, E): Podomere 3 curved, 3 times longer than wide, slightly longer than immovable finger (Fig. 11D). Hollowed-out inner margin with one lobe and four sclerotized spines, posterior aspect with *ca.* 29 small crenulated teeth. Immovable finger basally wide, apically tapering, 2.6 times longer than wide, strongly curved towards fixed finger. Podomere 1 and 2 with few setae on both sides (Fig. 11D), podomere 3 glabrous (Fig. 11E).

Female sexual characters: Second leg pair with well-developed coxal lobe.Vulva massive. Operculum well-rounded, protruding up to basal half of prefemur (Fig. 11F). Subanal shield almost divided into two, with strong invagination at apical margin (Fig. 11G). Washboard with three stridulation ribs on each side (Fig. 11G).

#### Etymology.

‘Lavasoa’, noun in apposition, after the Lavasoa (also called Ambatotsirongorongo) mountain, to which this species is endemic.

#### Distribution.

Endemic to the Lavasoa Mountain, where it could be recorded from two of the three remaining fragments. The species was common in the largest fragment of Grande Lavasoa, but only a single female could be collected at Petit Lavasoa.

### Sphaeromimus andohahela

Wesener sp. n.

http://zoobank.org/CE4D941A-88F1-4D02-B08D-D141F8BDC7E5

http://species-id.net/wiki/Sphaeromimus_andohahela

**Figure 13.**
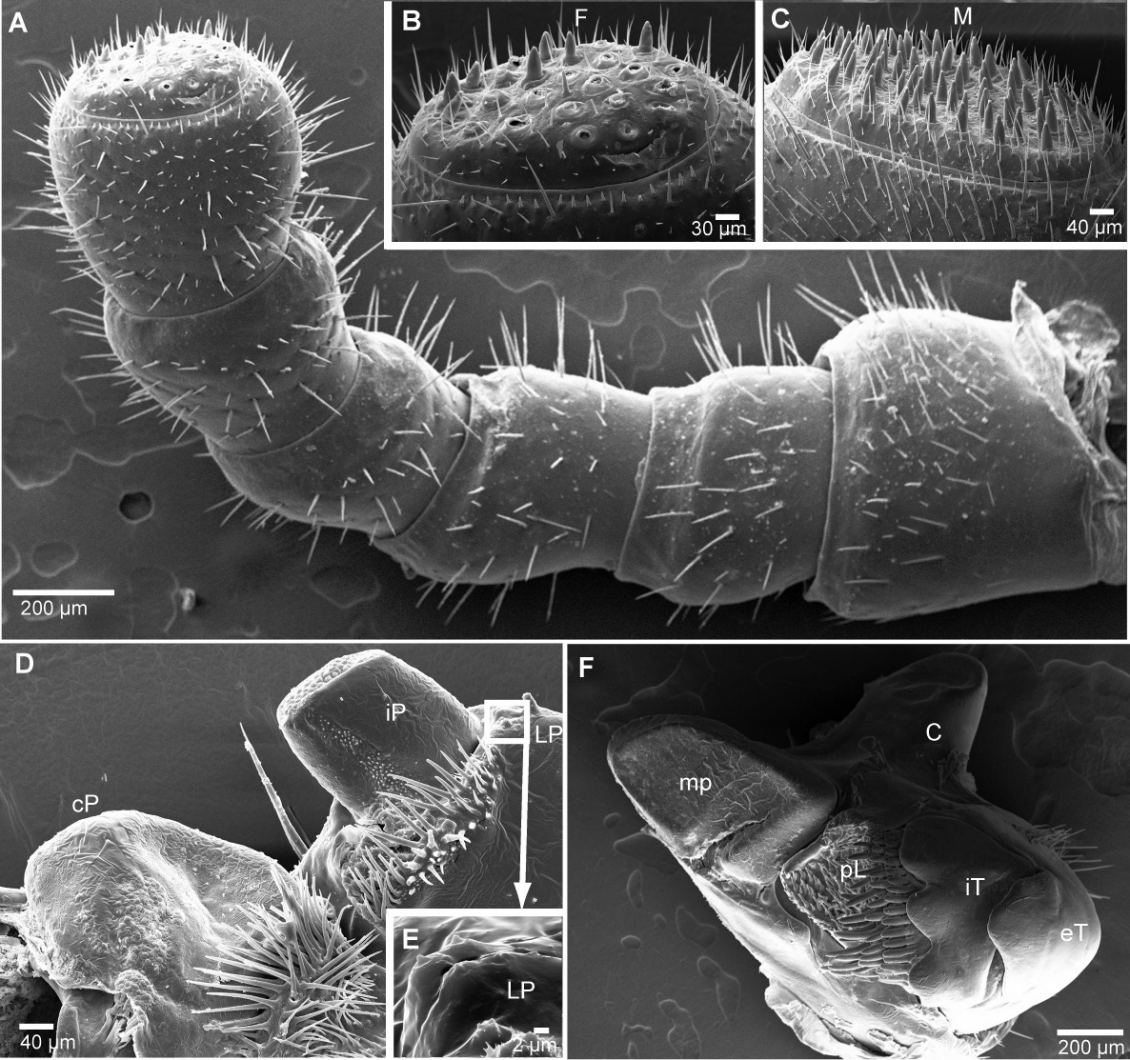
*Sphaeromimus andohahela* sp. n., **A, B, D–F** female paratype **C** male paratype, SEM. **A** right female antenna, lateral view **B** female antennomere 6 with disc **C** male antennomere 6 with disc **D** gnathochilarium, right corner, inner surface **E** rudimentary right lateral palpus ♀ left mandible, mesal view. Abbreviations: C = condylus; cP = central pads; eT = external tooth; iP = inner palpus; iT = combed inner tooth; LP = rudimentary lateral palpus; mp = molar plate; pL = pectinate lamellae.

**Figure 14.**
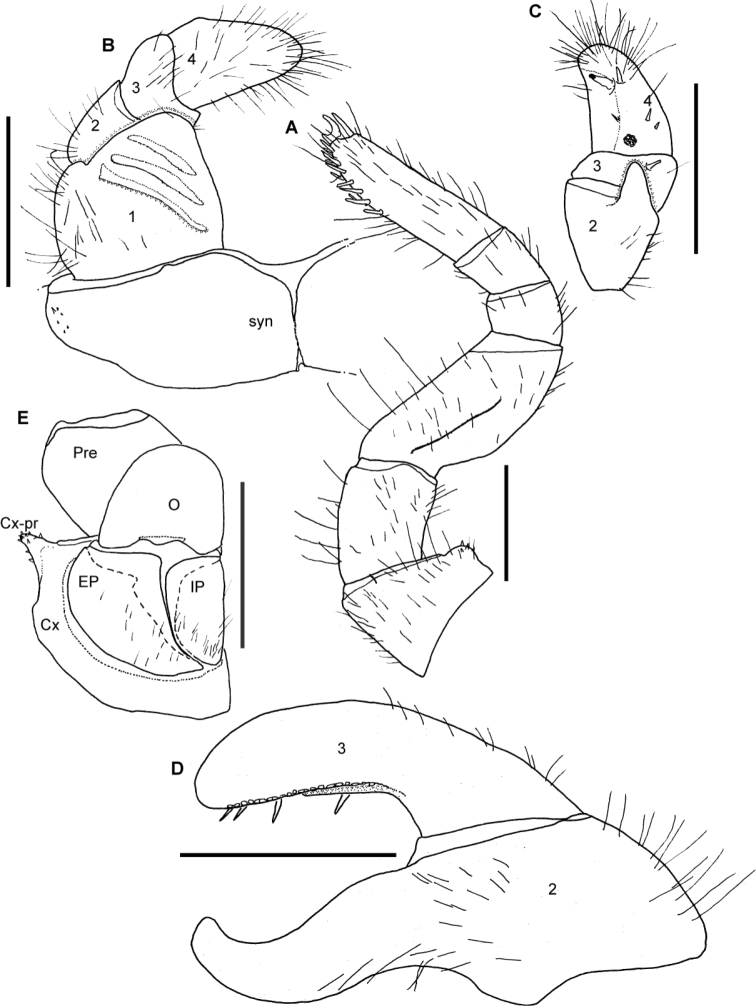
*Sphaeromimus andohahela* sp. n., **A–D** male holotype **E** female paratype. **A** left leg 9 **B** anterior telopod, anterior view **C** right anterior telopod, posterior view **D** left posterior telopod, posterior view **E** coxa and prefemur 2 with vulvae. Abbreviations: Cx = coxa; Cx-pr = coxal process; EP = external, lateral plate of vulva; IP = inner, mesal plate of vulva; O = operculum; Pre = prefemur; syn = syncoxite. Scale bars = 1 mm.

Figs 12B–D , 13 , 14 , 20

Sphaeromimus ‘sp. n. I Manantantely’; *Sphaeromimus* ‘sp. n. II Malio’:-- Wesener et al. 2010: 1185 (molecular phylogenetic analysis)

#### Material examined.

Type material. *Holotype*. 1 ♂, ZFMK MYR2322, Madagascar, Province Toliara, PN Andohahela, Isaka-Ivondro Nord, 24°46.302'S, 046°51.699'E, 571 m, rainforest, coll. Wesener & Schütte, 12.vi.2007.

**Paratypes.** 1 ♀, FMNH-INS 61135; 1 imm., FMNH-INS 61136; 1 ♀, FMNH 61137; 2 ♀, 2 imm., FMNH-INS 56212, all same data as holotype.

**Other material.** 6 ♂, ♀, Juv., FMNH-INS 56210, PN Andohahela, Malio, 24°55.810'S, 046°46.343'E, rainforest, coll. Wesener & Schütte, 30.v.2007; 1 ♂, ZFMK MYR2323, same data as previous; 9 ♂, ♀, imm., FMNH-INS 56209, Madagascar, Vohimena Chain, PR Manantantely, 24°59'17.14"S, 046°55'27.95"E, rainforest, coll. Wesener & Schütte, 06.vi.2007; 1 ♀, FMNH-INS 61140; 1 ♂, FMNH-INS 61132; 1 ♀, FMNH-INS 61138; 1 ♂, FMNH-INS 61139; 1 ♂, 2F, ZFMK MYR 2324, all same data as previous.

#### Diagnosis.

Small matte-black pill millipede with a dark brown head and collum and light brown appendages. Male harp with three stridulation ribs.

#### Description.

Measurements: male holotype: 20.2 long, 9.2 (2nd), 9.8 (8th) wide, 4.9 (2nd), 6.1 (10th = highest) high. Largest female (with eggs): 21.1 mm long, 9.6 mm (2nd), 10.75 (8th = widest) wide, 5.7 (2nd), 7.5 mm (10th = highest) high.

Colouration of tergites black, matte not shiny. Collum and head dark brown (Fig. 20). Depressions of paratergites as well as legs and antennae brownish.

Head: Eyes with >60 ocelli. Antennae quite long, protruding as far as leg 8. Antennomeres 1–5 with few setae, 6 densely pubescent (Fig. 13A). Antennomere 6 towards disc with single row of sensilla basiconica (Fig. 13B). Female with 54/56 (13B), male with 74/78 apical cones (Fig. 13C). Gnathochilarium typical for the genus (Fig. 13D), rudimentary lateral palpi with a field of four sensory cones (Fig. 13E). Mandible with the typical shape of the order, inner tooth 3-combed, with six long pectinate lamellae, condylus with a sharp and quite large groove at its apex (Fig. 13F).

Collum glabrous except few setae at its margin.

Thoracic shield smooth and glabrous, few setae in grooves. Tergites 3–12 smooth, but not glossy, paratergite tips of midbody tergites only weakly projecting posteriorly (Fig. 20).

Anal shield massive, well-rounded, lacking pubescent area.

Endotergum inner section with numerous short triangular spines and very few setae (Fig. 12B). Between ridge and inner area two rows of weakly impressed, circular cuticular impressions. Externally two irregular rows of marginal bristles (Fig. 12B–D). Bristles short, barely protruding up to tergite margin.

First stigma-carrying plate with a well-rounded apex.

Leg 1 with 2 to 4, 2 with 5 or 6, 3 with 10 or 11 ventral spines. Leg pairs 4–21 with 12–14 ventral spines. Coxa process weakly developed (Fig. 14A). Femur 2, tarsus 2.9 times longer than wide.

Male gonopore inconspicuous.

Anterior telopod (Fig. 14B, C): Harp carrying three stridulation ribs (Fig. 14B). Shape usual for the genus, telopoditomere 4 as long as 2 and 3 combined, with one large triangular spine and 4 smaller ones (Fig. 14C). Telopoditomere 3 with a spine juxtaposed to process of telopoditomere 2 (Fig. 14C).

Posterior telopod (Fig. 14D): Podomere 3 curved, 3 times longer than wide, slightly longer than immovable finger (Fig. 14D). Hollowed-out inner margin with one lobe and four sclerotized spines, posterior aspect with *ca.* 21 small crenulated teeth. Immovable finger basally wide, apically tapering, 3.1 times longer than wide, strongly curved towards fixed finger. Podomere 1 and 2 with few setae on both sides (Fig. 14D), podomere 3 with a few setae at its margins.

Female sexual characters: Second leg pair with well-developed coxal lobe. Vulva massive. Operculum well-rounded, protruding above basal half of prefemur (Fig. 14E). Subanal shield almost divided into two, with strong invagination at apical margin. Washboard with three stridulation ribs on each side.

Intraspecific variation: The endotergum differs slightly in the development of the cuticular impressions between the specimens from Isaka-Ivondro (Fig. 12B), Malio (Fig. 12C) and Manantantely (Fig. 12D). The specimens from the three localities also differ slightly in their colour pattern: In specimens from Manantantely and Isaka-Ivondro, the head and collum are dark brown, while those from Malio have a brown thoracic shield too.

Genetic distances in the COI gene between the three populations are 2.9–4%, while even the two individuals from Malio show a variation at the population level of 3.6%. Future studies involving more localities and specimens should investigate whether or not gene flow occurs between the different populations of *Sphaeromimus andohahela*.

#### Etymology.

‘andohahela’, noun in apposition, after the type locality, the rainforests of the national park Andohahela.

#### Distribution.

Widespread in the lowland and montane rainforests of the northern Anosy and Vohimena mountain chains.

### Sphaeromimus ivohibe

Wesener sp. n.

http://zoobank.org/E8220C56-2B68-414F-8949-27E207D3B0C2

http://species-id.net/wiki/Sphaeromimus_ivohibe

Figs 15 , 16A

#### Material examined.

Type material. *Holotype*. 1 ♂, FMNH-INS 8184, Madagascar, Province Fianarantsoa, extreme northern limit of Réserve Speciale de Ivohibe, along Hefitany Riv., ca. 7.5 km ENE Ivohibe, 22.4700°S, 46.9600°E, 1200 m, coll. S. M. Goodman, 03.–09.ix.1997.

#### Diagnosis.

Small shining black pill millipede with orange-reddish appendages. Of all currently known *Sphaeromimus* with highest number of stridulation ribs, 6, on male harp. Posterior telopods unique, with two large membranous lobes.

#### Description.

Measurements: male holotype: 20.6 long, 8.6 (2nd), 9.05 (8th = widest) wide, 5.2 (2nd), 6.1 (8th = highest) height.

Colouration of tergites shining black. Paratergite impressions and groove of thoracic shield dark greenish. Legs, antennae and pleurites orange-red, eyes green.

Head: Eyes with >60 ocelli. Antennae very short, protruding to coxa 5. Antennomeres 1–4 with few longer setae, 5 and 6 densely pubescent. Antennomere 6 towards disc with single row of sensilla basiconica. Male with 34/35 apical cones. Mouthparts not dissected.

Collum glabrous except few setae at margins.

Thoracic shield smooth and glabrous, few setae in grooves. Grooves deep. Tergites 3–12 smooth, except for paratergite depressions. Paratergite tips of midbody tergites weakly projecting posteriorly.

Anal shield well-rounded, lacking pubescent area.

Endotergum inner section with numerous short triangular spines and long setae (Fig. 16A). Between ridge and inner area two rows of weakly impressed, circular cuticular impressions. Externally single, sparse row of marginal bristles (Fig. 16A). Bristles short, not protruding up to tergite margin.

**Figure 15.**
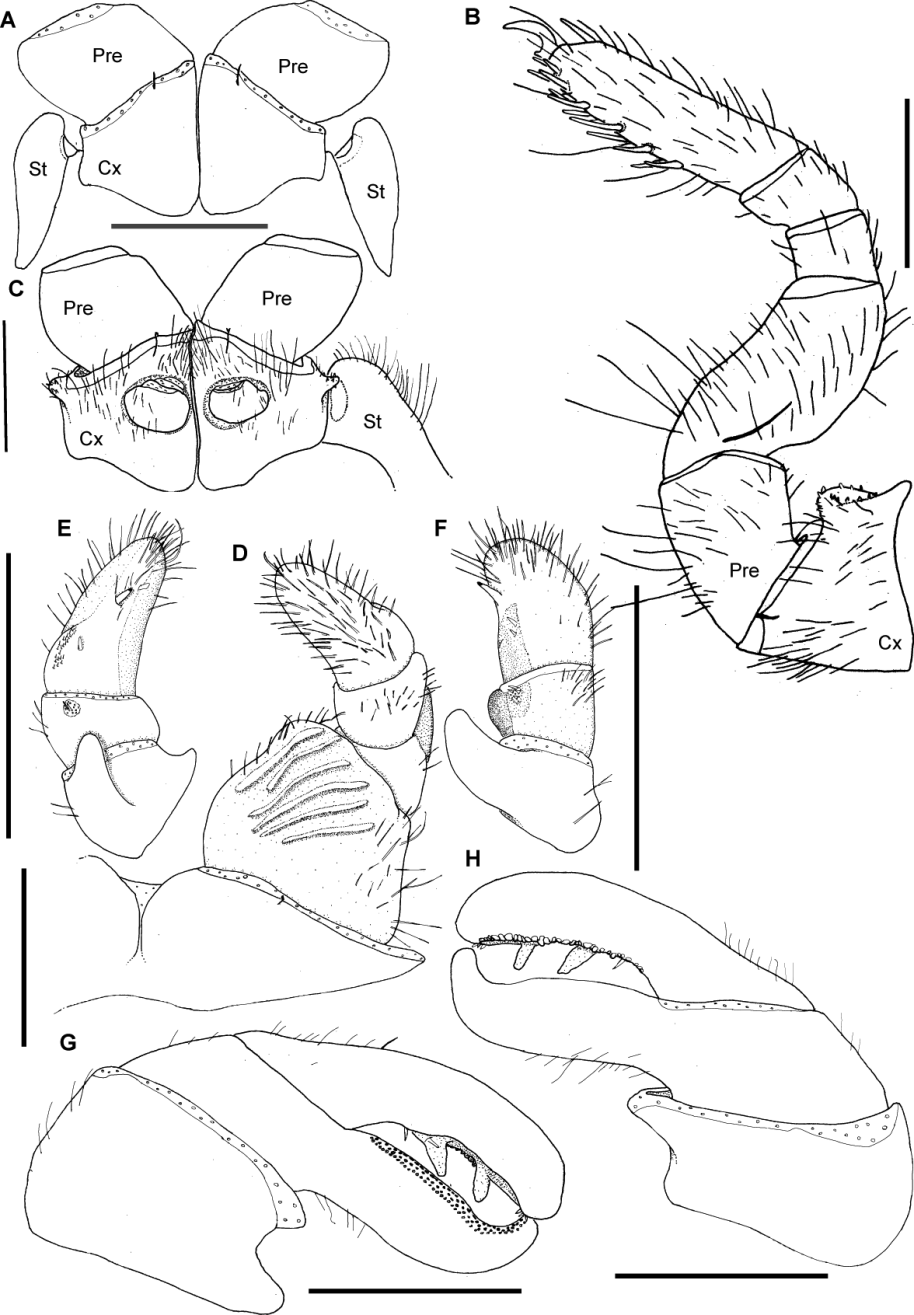
*Sphaeromimus ivohibe* sp. n., holotype. **A** coxae and prefemora 1 with stigmatic plates **B** left leg 9 **C** coxae and prefemora 2 with gonopore and stigmatic plate **D** right anterior telopod, anterior view **E** right anterior telopod, posterior view ♀ right anterior telopod, lateral view **G** left posterior telopod, anterior view **H** left posterior telopod, posterior view. Abbreviations: Cx = coxa; Pre = prefemur; St = stigmatic plate. Scale bars = 1 mm.

First stigma-carrying plate with a well-rounded projecting apex (Fig. 15A).

Leg 1 with 2, 2 with 3, 3 with 8 ventral spines. Leg pairs 4–21 with 12 ventral spines. Coxa process visible, but not as well developed as those of anterior legs (Fig. 15B). Femur 1.6, tarsus 2.9 times longer than wide (Fig. 15B).

**Figure 16.**
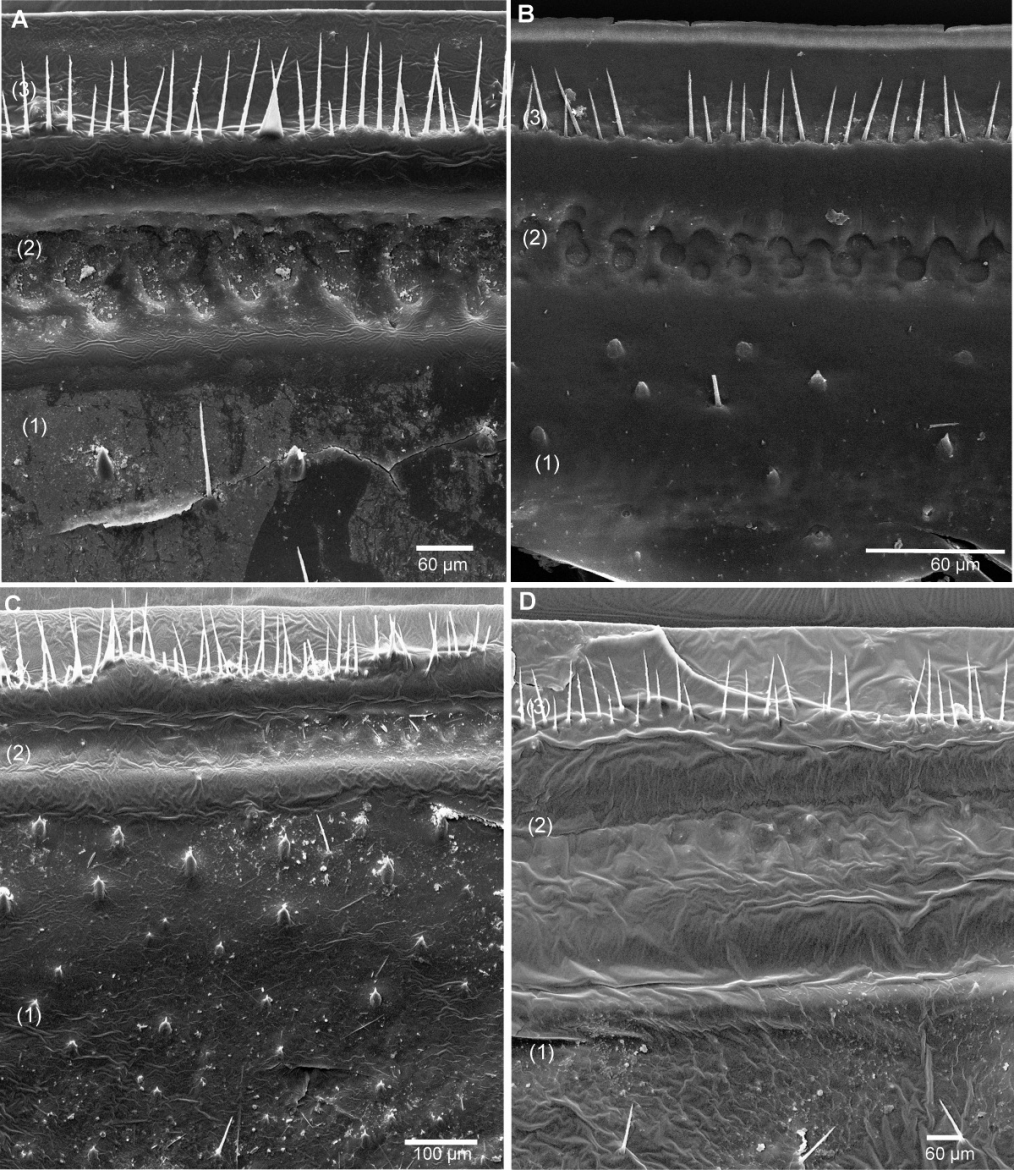
SEM, Endoterga of mid-body tergite. **A** *Sphaeromimus ivohibe* sp. n., paratype **B** *Sphaeromimus saintelucei* sp. n., holotype from Isaka-Ivondro **C** *Sphaeromimus andrahomana* sp. n., holotype **D** *Sphaeromimus andrahomana* cave specimen. Abbreviations: (1) = inner area with large spines and long setae; (2) = area with cuticular patterns; (3) = outer area with row(s) of marginal bristles and tergite margin.

Male gonopore typical for the genus (Fig. 15C).

Anterior telopod (Fig. 15D–F): Harp carrying six stridulation ribs (Fig. 15D). Shape usual for the genus, telopoditomere 4 longer than 2 and 3 combined, with one large triangular spine and 2 or 3 smaller ones (Fig. 15E, F), laterally with a field of sensory hair (Fig. 15E). Podomere 3 with several small spines juxtaposed to process of telopoditomere 2.

Posterior telopod (Fig. 15G, H): Podomere 3 straight, 4.4 times longer than wide, slightly longer than immovable finger (Fig. 15G). Hollowed-out inner margin with two lobes and four sclerotized spines, posterior aspect with *ca.* 36 small crenulated teeth (Fig. 15H). Immovable finger apically only weakly tapering, only its apex curved towards podomere 3. Podomere 1–3 glabrous except for a few marginal hair with few setae (Fig. 15G, H).

Female unknown.

#### Etymology.

‘ivohibe’, noun in apposition, after the type locality, the national park Ivohibe.

#### Distribution.

Only known from the type locality.

### Sphaeromimus saintelucei

Wesener sp. n.

http://zoobank.org/EE026627-2CE4-4492-8141-9A841E794635

http://species-id.net/wiki/Sphaeromimus_saintelucei

Figs 16B , 17

#### Material examined.

Type material. *Holotype*: 1 ♂, ZFMK MYR889, Madagascar, Province Toliara, Sainte Luce, fragment S8, 24°46.520'S, 047°09.074'E, 28 m, littoral forest on basaltic soil, coll. Wesener & Schütte, 29.v.2007.

**Paratypes.** 1 ♂, FMNH-INS 61089, same data as holotype; 1 ?, FMNH-INS 61088, same data as holotype.

#### Diagnosis.

*Sphaeromimus saintelucei* sp. n. shares its small size (<20 mm), total absence of a coxal lobe at midbody legs in combination with slender posterior telopods of which the apex of the immovable finger is strongly curved only with *Sphaeromimus inexpectatus* Wesener & Sierwald, 2005. *Sphaeromimus saintelucei* differs from the latter in the dull brown colour (shiny pink in *Sphaeromimus inexpectatus*), and the anterior telopods. Both species differ genetically at 4–4.8% of their COI gene.

#### Description.

Measurements: male holotype: 15.8 long, 6.6 (2nd) wide, 4.1 (2nd) high.

Colouration of tergites dull brown. Paratergite impressions and groove of thoracic shield slightly lighter. Legs, antennae and pleurites orange-red, eyes green.

Head: Eyes with >45 ocelli. Antennae short, protruding to coxa 4. Antennomeres 1–5 with few longer setae, 6 densely pubescent. Antennomere 6 huge, large than 3 basal antennomere combined, towards disc with single row of sensilla basiconica. Male with 49/48 apical cones. Mouthparts not dissected.

Collum glabrous except few setae at margins.

Thoracic shield smooth and glabrous, few setae in grooves. Grooves deep. Tergites 3–12 smooth, except for paratergite depressions. Paratergite tips of midbody tergites weakly projecting posteriorly.

Anal shield well-rounded, lacking pubescent area.

Endotergum inner section with few short triangular spines and long setae (Fig. 16B). Between ridge and inner area two rows of weakly impressed, circular cuticular impressions. Externally single, extremely sparse row of marginal bristles (Fig. 16B). Bristles short, not protruding up to tergite margin.

First stigma-carrying plate with a well-rounded not-projecting apex.

**Figure 17.**
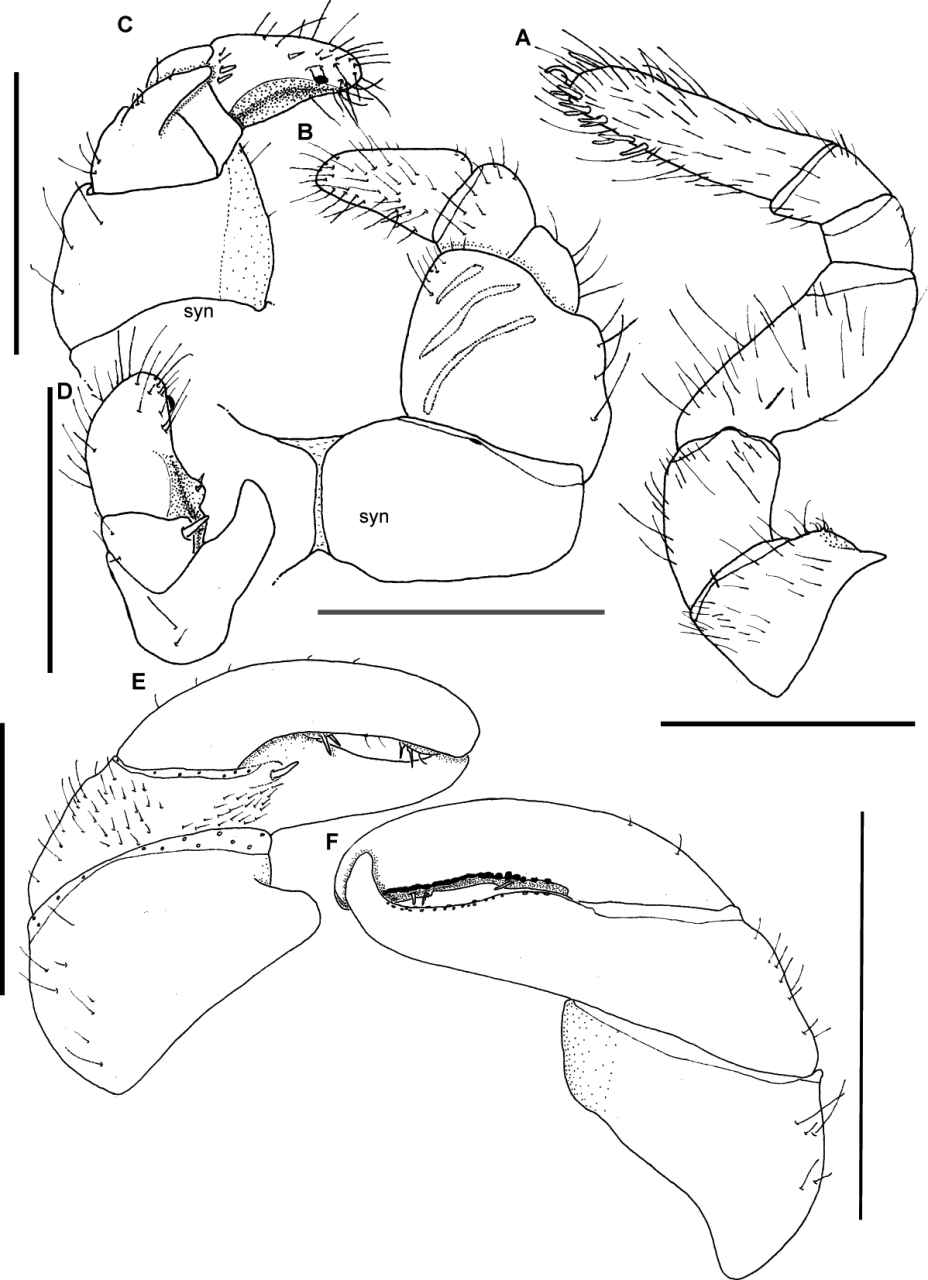
*Sphaeromimus saintelucei* sp. n., holotype. **A** left leg 9 **B** right anterior telopod, anterior view **C** left anterior telopod, posterior view **D** right anterior telopod, lateral view **E** left posterior telopod, anterior view ♀ left posterior telopod, posterior view. Abbreviations: syn = syncoxite. Scale bars = 1 mm.

Leg 1 with 2, 2 with 2, 3 with 4 or 5 ventral spines. Leg-pairs 4–21 with 7–10 ventral spines. Coxa process invisible (Fig. 17A), only weakly developed at anterior legs. Femur 2, tarsus 3.2 times longer than wide (Fig. 17A).

Male gonopore typical for the genus.

Anterior telopod (Fig. 17B–D): Harp carrying three stridulation ribs (Fig. 17B). Shape usual for the genus, telopoditomere 4 as long as 2 and 3 combined, with one large triangular spine and 2 or 3 smaller ones (Fig. 15C, D). Podomere 3 with one large spine juxtaposed to process of telopoditomere 2 (Fig. 17D).

Posterior telopod (Fig. 17E, F): Podomere 3 weakly curved, 4.1 times longer than wide, longer than immovable finger (Fig. 17E). Hollowed-out inner margin with single lobe and four sclerotized spines, posterior aspect with *ca.* 24 small crenulated teeth (Fig. 17F). Immovable finger apically strongly tapering, its apex strongly curved and overlapping podomere 3. Immovable finger with single spine at its base (Fig. 17E). Podomere 1 and 3 glabrous except for a few marginal hair with few setae, podomere 2 on posterior side glabrous (Fig. 17F), on anterior side with several hairs (Fig. 17E).

Female unknown.

#### Etymology.

‘saintelucei’, adjective, after the type locality, and only area of occurrence, the littoral rainforest of Sainte Luce.

#### Distribution.

Only known from the only remaining southern lowland forest on basaltic soil, the tiny fragment S8 of Sainte Luce. In the nearby fragments on sandy soil, *Sphaeromimus splendidus* occurs.

### Sphaeromimus andrahomana

Wesener sp. n.

http://zoobank.org/DD6E47B7-56DB-44FB-8870-9380460B13F6

http://species-id.net/wiki/Sphaeromimus_andrahomana

**Figure 18.**
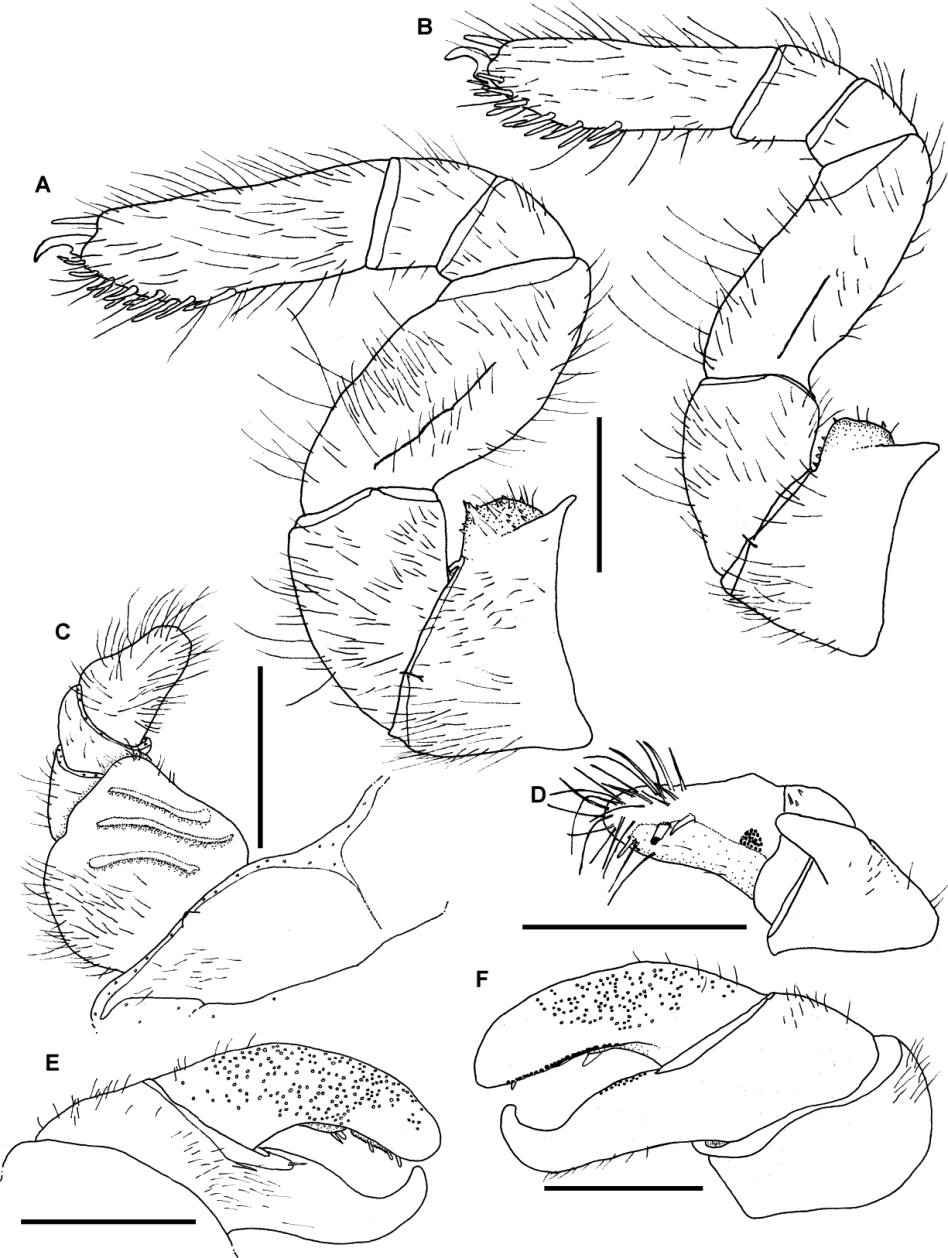
*Sphaeromimus andrahomana* sp. n.. **A** left leg 9, holotype **B** left leg 9, cave specimen **C** anterior telopod, anterior view **D** right anterior telopod, posterior view **E** left posterior telopod, anterior view ♀ left posterior telopod, posterior view. Scale bars = 1 mm.

Figs 1C , 16C, D , 18

Sphaeromimus ‘sp. n. V Grotte’: Wesener et al. 2010: 1185 (molecular phylogenetic analysis)

#### Material examined.

Type material. *Holotype*. 1 ♂, FMNH-INS 562214, N of village of N of Ankapaky, close to the Grotte d’Andrahomana, 25°11'18.87"S, 46°38'45.14”E, 70 m, dry forest plateau with deep ravines, coll. Wesener & Schütte, 20.v.2007

**Other material examined.** 1 ♂, FMNH-INS 56211, Grotte Andrahomana, 24°51.006'S, 046°55.907'E, inside humid cave, coll. Wesener & Schütte, 20.v.2007.

#### Diagnosis.

Small matte-black pill millipede with a dark brown head and collum. Similar to *Sphaeromimus andohahela* but differs from the latter in weakly developed cuticular patterns and presence of numerous small pits on movable finger of posterior telopod. Genetical distance of the COI gene between both species is 10–11.4%.

#### Description.

Measurements (holotype): 21.1 mm long, 9.8 mm (2nd), 10.7 mm (8th - widest) wide, 5.5 (2nd), 7.0 mm (10th, highest) height.

Colouration of tergites black, collum and head brown. Paratergite impressions light brown to olive-greenish, legs and antennae olive green (faded to white in ethanol), pleurites light brown, eyes green.

Head: Eyes with >55 ocelli. Antennae short, posteriorly protruding to coxa 5. Antennomeres 1–5 with few longer setae, 6 densely pubescent. Antennomere 6 towards disc with single row of sensilla basiconica. Male with 58/61 apical cones. Mouthparts not dissected.

Collum glabrous except few setae at margins.

Thoracic shield smooth and glabrous, few setae in grooves. Grooves deep. Tergites 3–12 smooth, except for paratergite depressions. Paratergite tips of midbody tergites weakly projecting posteriorly.

Anal shield well-rounded, lacking pubescent area.

Endotergum inner section with few short triangular spines and long setae (Fig. 16C). Between ridge and inner area two rows of weakly impressed, circular cuticular impressions. Externally two dense rows of marginal bristles (Fig. 16C). Bristles long, protruding above tergite margin.

First stigma-carrying plate with a well-rounded not-projecting apex.

Leg 1 with 2 or 3, 2 with 5 or 6, 3 with 10 or 11 ventral spines. Leg pairs 4–21 with 12–14 ventral spines. Coxa process well developed (Fig. 18A), only weakly developed at anterior legs. Femur 1.6, tarsus 3.0 times longer than wide (Fig. 18A).

Male gonopore typical for the genus.

Anterior telopod (Fig. 18C, D): Harp carrying three stridulation ribs (Fig. 18C). Shape usual for the genus, telopoditomere 4 as long as 2 and 3 combined, with one large triangular spine and 2 or 3 smaller ones (Fig. 18C, D). Podomere 3 with three small spines juxtaposed to process of telopoditomere 2 (Fig. 18D).

Posterior telopod (Fig. 18E, F): Podomere 3 weakly curved, 3 times longer than wide, longer than immovable finger (Fig. 18E). Both sides with conspicuous pits (Fig. 18E, F). Hollowed-out inner margin with single lobe and five sclerotized spines, posterior aspect with *ca.* 24 small crenulated teeth (Fig. 18F). Immovable finger apically strongly tapering, its apex strongly curved and overlapping podomere 3. Podomere 1 and 3 glabrous except for a few marginal hair with few setae, podomere 2 on posterior side glabrous (Fig. 18F), on anterior side with several hair (Fig. 18E).

Female unknown.

Etymology: ‘andrahomana’, noun in apposition, after the famous cave close to the type locality, the Grotte d’Andrahomana.

#### Distribution.

Relic occurrence in the Grotte D’Andrahomana. The single individual found close to the village Ankapaky might be an indication of a more widespread occurrence in the little explored Vohisandria and Amboalaingo hills N. of Ankapaky and S. of Ranopiso.

#### Discussion.

Intraspecific variations: The cave specimen shows a distinct colour pattern: tergites very light brown with dark brown posterior margins (Fig. 1C), head and collum light brown, legs and antenna, at least apically red. The endotergum shows fewer spines and hair in the cave specimen (Fig. 16D) when compared to the holotype (Fig. 16C). Furthermore, the leg of the cave specimen is distinctively slenderer at its basal joints (Fig. 18B), the femur being 2.2 (holotype 1.6), the tarsus 3 (holotype 3) times longer than wide. Despite their large morphological difference show both specimens the same COI haplotype.

### Undetermined *Sphaeromimus* spp. records

**Material examined.** 2 ♀, CAS ENT 9032816, Madagascar, Vevembe, Farafangana, Province Fianarantsoa, Forêt de Vevembe, 66.6 km 293°WNW Farafangana, 22°47'28"S, 047°10'55"E, 600 m, rainforest transitioning to montane forest, coll. B.L. Fisher et al., 23.iv.2006, general collecting; 1 ♀, MNHN ‘11’, Madagascar, Ikongo, coll. G. Grandidier, 21.V.1901, potential locality: Province Fianarantsoa, Fort Carnot, 21°51'30"S, 47°26'30’’ E (similar to the type locality of *Sphaeromimus vatovavy*); 1 ♂ (broken), MNHN ‘53’, Madagascar, Cap Diego, coll. R. Decary, Aug.-Sept. 1916, potential locality: Diego-Suarez (Antsiranana)?; 4 ♀ (together with 2 *Zoosphaerium libidinosum*), MNHN ‘114’, Madagascar, envoi nºVI, ‘Glomeris’, coll. G. Petit, entree 24-1922.

### Updated distribution of *Sphaeromimus*:

**Figure 19.**
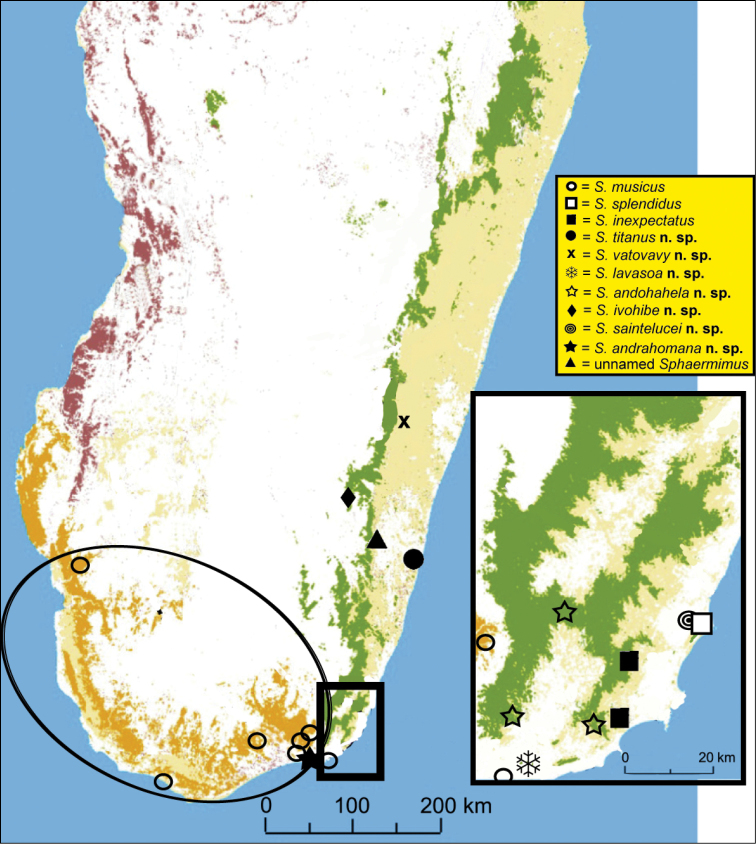
Distribution map of the genus *Sphaeromimus*. Map and vegetation types modified after Thiele et al. 2013.

Despite the description of seven new species and numerous additional localities *Sphaeromimus* is still restricted to southeastern Madagascar (Fig. 19). Most species occur in the rainforest and littoral rainforest, with only the widespread *Sphaeromimus musicus* occurring in the spiny forest ecosystem. *Sphaeromimus* specimens could be discovered in every single explored southeastern Malagasy rainforest (Fig. 19), always in sympatry with species of the other Malagasy genus of giant pill-millipedes, *Zoosphaerium* (see Wesener 2009).

### Genetic distances between *Sphaeromimus* species

**Figure 20.**
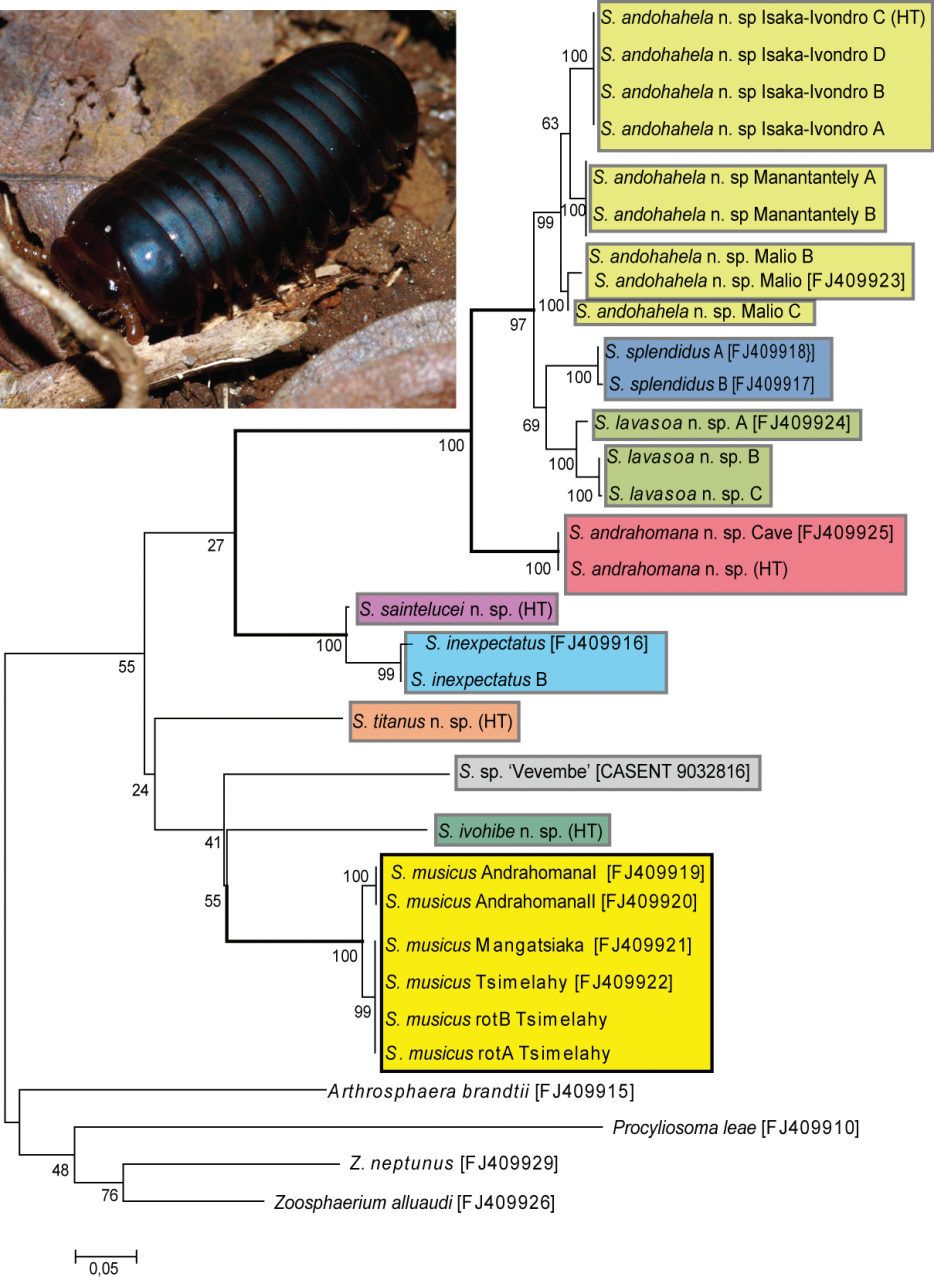
Maximum likelihood tree obtained from the COI dataset after 1000 bootstrap replicates under the GTR+I+G model. Habitus photograph shows *Sphaeromimus andohahela* from Manantantely. Colours used to separate species. Green colours = mid-elevation rainforest; Blue & Red colours, littoral and lowland rainforests; Yellow colours = southern spiny forest. See table 1 for more details about sequenced specimens.

The analysis of the barcoding fragment of the COI gene provided a good resolution at the species level; all *Sphaeromimus* species are monophyletic and form well-supported terminals (Fig. 20). Genetic distances between the different *Sphaeromimus* species is 4.0% (*Sphaeromimus inexpectatus* and *Sphaeromimus saintelucei*) but mostly between 8–20% and up to 25.3% (*Sphaeromimus musicus* and *Sphaeromimus splendidus*). Based on their mitochondrial DNA, the species of the genus can be separated into two groups, albeit without any statistical support: (1) the extreme southeastern humid forest clade, and (2) a clade incorporating the spiny forest *Sphaeromimus musicus* together with the slightly more northern rainforest species from Ivohibe, Vevembe and Manombo (Fig. 20).

In the latter clade, all species show high genetic distances of 16–21% to one another and no sub-grouping receives any statistical support. More structure can be observed in the extreme southeastern clade. *Sphaeromimus inexpectatus* from the littoral rainforest of Mandena and the rainforest of Enato forms a well-supported monophyly with *Sphaeromimus saintelucei* from the nearby littoral rainforest fragment on basaltic soil at Sainte Luce S8. This monophyly is juxtaposed to a clade comprising *Sphaeromimus andrahomana*, *Sphaeromimus lavasoa*, *Sphaeromimus splendidus*, and *Sphaeromimus andohahela*. Within this clade, *Sphaeromimus andrahomana*, the southern-most taxon, is in the basal-most position differing by more than 10% of its base pairs from any other *Sphaeromimus* species (Fig. 20). *Sphaeromimus andohahela* from the Andohahela and Vohimena mountains is sister to a clade comprising the well-supported (69%) *Sphaeromimus splendidus* from the littoral rainforest sandy soil fragment S9 at Sainte Luce and *Sphaeromimus lavasoa* from the southern isolated Lavasoa Mountain.

## Discussion

### Incorporating COI barcode data into the taxonomy of *Sphaeromimus*

Genetic distances between the species of *Sphaeromimus* are high, hinting at an old age of the speciation events shaping the current species of the genus (see also Wesener et al. 2010). This presumably old age is further highlighted by the fact that the deeper branches receive very little statistical support (Fig. 20). The COI gene probably already lost its resolution because too many reverse substitutions occurred.

Nevertheless, the COI gene is a powerful taxonomic tool, greatly improving our systematic understanding and has led to the description of new species in the family Zephroniidae from Asia (Wongthamwanich et al. 2012, Golovatch et al. 2012), and here also in the Malagasy genus *Sphaeromimus*. The incorporation of the COI barcoding gene allows a better separation of the small black taxa in the extreme southeastern clade which are more difficult to distinguish (Fig. 20). The COI data further led to the direct discovery of a pseudo-cryptic species, *Sphaeromimus saintelucei*. The few obtained *Sphaeromimus* specimens from the heavily degraded littoral rainforest fragment S8 at Sainte Luce (Fig. 19) were first mistaken for juveniles of *Sphaeromimus splendidus*. Only the very high genetic distances observed prompted a more close morphological study, which confirmed a closer morphological similarity with *Sphaeromimus inexpectatus*, matching the results from the analysis of the COI gene (Fig. 20). Additionally, the different colour morphs of *Sphaeromimus musicus* (Fig. 1A) could be correctly determined as just that, based on their identical COI sequences. The cave specimen of *Sphaeromimus andrahomana*, quite unusual in its colouration (Fig. 1C) and also morphology (Fig. 18B) would have been described as a separate species if not for the 0% difference in its COI sequence with those of the holotype of *Sphaeromimus andrahomana* (Fig. 20).

The interesting relationships and biogeographic patterns among the species of *Sphaeromimus*, with species like *Sphaeromimus splendidus* and *Sphaeromimus saintelucei* occurring in close proximity to one another (Fig. 19), but showing great genetic distances (21.7%) and no close relationship (Fig. 20), are further hints to the interesting biogeographic mechanisms shaping the current distribution of *Sphaeromimus* species in southeastern Madagascar. These patterns should be studied further using more molecular markers.

## Supplementary Material

XML Treatment for Sphaeromimus

XML Treatment for Sphaeromimus musicus

XML Treatment for Sphaeromimus splendidus

XML Treatment for Sphaeromimus inexpectatus

XML Treatment for Sphaeromimus titanus

XML Treatment for Sphaeromimus vatovavy

XML Treatment for Sphaeromimus lavasoa

XML Treatment for Sphaeromimus andohahela

XML Treatment for Sphaeromimus ivohibe

XML Treatment for Sphaeromimus saintelucei

XML Treatment for Sphaeromimus andrahomana
